# SARS-CoV-2 nucleocapsid protein forms complexes with soluble complement regulatory proteins that can bind to the virion

**DOI:** 10.1038/s41598-026-37866-4

**Published:** 2026-01-29

**Authors:** Jakub Víglaský, Katarína Bhide, Lea Talpasova, Ľubica Fialová, Mangesh Bhide

**Affiliations:** 1 Laboratory of Biomedical Microbiology and Immunology, University of Veterinary Medicine and Pharmacy in Košice, Komenského 73, Košice, 041 81 Slovakia; 2 Institute of Neuroimmunology, Slovak Academy of Sciences v. v. i, Dúbravská cesta 9, 845 10 Bratislava, Slovakia

**Keywords:** SARS-CoV-2, Complement regulatory proteins, Nucleocapsid protein, Spike protein, Complement system evasion, Biochemistry, Diseases, Immunology, Microbiology, Molecular biology

## Abstract

The SARS-CoV-2 nucleocapsid protein has been detected in the plasma of COVID-19 patients, and its levels in the plasma correlate with the severity of the disease. It is also an immunomodulatory protein, triggering the release of proinflammatory cytokines. Complement system dysregulation in COVID-19 patients led us to hypothesize that either nucleocapsid protein or spike protein might interact with the proteins of the complement system, mainly complement regulatory proteins (CRPs). We demonstrate that the nucleocapsid protein, but not the spike protein, binds to multiple CRPs, including C1-inhibitor, C4-binding protein, factor H, and vitronectin. The nucleocapsid protein binds to both the recombinant spike protein and the SARS-CoV-2 virions. We further demonstrated that the virion-nucleocapsid-CRP complex could be formed. Recruitment of the CRPs on SARS-CoV-2 virion mediated by nucleocapsid protein deserves further investigation to reveal complement modulation strategies of SARS-CoV-2.

**Supplementary Information:**

The online version contains supplementary material available at 10.1038/s41598-026-37866-4.

## Introduction

 Co-evolution of viruses and their hosts has led to a variety of beneficial adaptations such as the production of viral immunomodulatory proteins (VIPs), which interact with the host’s innate and adaptive immune systems. VIPs are either derived from host proteins (e.g., vIL-10 protein of Epstein-Barr virus) or do not possess any sequence homology to host proteins (e.g., smallpox inhibitor of complement enzymes of Variola virus)^1,2^. Viral immunomodulatory proteins can suppress host signaling molecules, such as the cytokine-binding proteins M3 of herpesvirus or proteases, such as the viral serpin Serp-1 of myxoma virus. They can also facilitate viral infection by increasing infectivity, such as the HIV-1 Tat protein, which binds to the cell surface and increases the expression of HIV-1 co-receptors^3^. Some of the unique VIPs, the viral complement inhibitors, are utilized by viruses to evade the first line of host defense—the complement system^4^.

The complement system, a canonical innate immune system, consists of over 30 plasma proteins and associated regulatory elements that recognize and effectively neutralize invading bacteria, parasites, and viruses. Activation of the complement cascade leads to pathogen neutralization and infected cell lysis^5^. The complement system is tightly controlled by complement regulatory proteins (CRPs) that distinguish between “self” and “non-self” cells and defend host cells by inhibiting the activation of the complement system on their surface^6^. Many pathogens have evolved complement evasion mechanisms in which they bind one or more CRPs to their surface and act as if they are the “self” entity. Exploitation of the CRPs by bacteria to evade complement-mediated lysis is well studied. We previously demonstrated that several *Borrelia* strains express surface proteins with affinity for the soluble CRPs such as C4 binding protein (C4BP), factor H (fH), and vitronectin (VTN), and some *Francisella tularensis subsp. holoarctica* strains bind VTN. The binding of CRPs on the surface of pathogens blocks the complement cascade^7^. For example, fH on the pathogen surface protects it from activation of the alternative pathway by accelerating the decay of C3 convertase and acting as a cofactor for factor I to cleave C3. C4BP binding inhibits the classical and lectin pathways by accelerating the decay of C3 convertases on the pathogen surface and factor I-mediated cleavage of C4b and C3b. Binding of fH and/or C4BP also reduces opsonization and phagocytosis^8,9^. VTN recruited by bacteria inhibits the formation of membrane attack complex (MAC), thereby protecting the pathogen from complement-mediated lysis^10^.

Viruses have also evolved unique strategies for evading the host complement system, such as the production of virus-encoded proteins that mimic host complement control protein (CCP) domains or secreted proteins that bind to host CRPs. For example, vaccinia virus induces secretion of 35 kDa viral complement control protein, which contains CCP domains and structurally resembles host C4BP^11^. The viral complement control protein protects the virus and infected cells by limiting complement activation by dissociating C3 and C5 convertases and acting as a cofactor for factor I-mediated inactivation of C3b and C4b^12^. The nonstructural protein 1 (NS1) of various flaviviruses is a prominent protein secreted by infected cells that disrupts the complement cascade. The secreted form of NS1 binds to the surface of epithelial and mesenchymal cells and has been shown to bind soluble CRPs, including fH, C4BP, VTN, and clusterin^13^. It was demonstrated that interaction between NS1 and fH or C4BP enhances factor-I-mediated cleavage of C3b and C4b^14,15^. NS1 can recruit soluble CRPs to the surface of infected cells, protecting them from complement attack^14,15^. NS1 can also recruit VTN on the surface of infected cells, inhibiting the polymerization of C9 and providing protection from complement-mediated cell lysis^16^. SARS-CoV-2 employs a similar strategy with its secreted ORF8 protein, which binds to C3b and thus inhibits pro-C3-convertase formation, leading to complement evasion^17^.

Some viruses incorporate membrane-associated CRPs (such as CD59, CD46, and CD55) into their envelope or attract soluble CRPs on the surface. For example, the hepatitis C virus incorporates CD59 in its envelope [18], the parainfluenza virus 5 incorporates CD46 and CD55 [19], and HIV-1 incorporates CD46, CD55, and CD59 [20, 21]. HIV-1 is also able to attract soluble fH on its surface via gp120 and gp41 proteins^18^. Virion-based strategies are also employed by Nipah and Chikungunya viruses, where virions have factor I-like protease activity^19,20^. In the case of SARS-CoV-2, similar complement evasion mechanisms have been suggested. For example, it has been proposed that the SARS-CoV-2 virus can incorporate membrane-associated regulators like CD55 and CD59, as well as bind soluble fH via its membrane protein^21^.

Several studies have attempted to reveal excessive complement activation and evasion strategies of SARS-CoV-2^22–24^. It was proposed that cells infected with SARS-CoV-2 release nucleocapsid (N) protein, which can activate the lectin pathway by binding directly to mannan-binding lectin-associated serine protease 2 (MASP-2), resulting in excessive complement activation^22^. However, recent studies have challenged this by failing to replicate lectin pathway activation^23,24^, which could be due to different sources of recombinant N protein, emphasizing the importance of additional research in the field of N protein and its interactions with the complement system. N protein is the most abundant structural protein, essential for viral genome packaging^25^. It is encoded by open reading frame 9 of the viral genome and spans 419 amino acids. The N protein has five distinct domains, three intrinsically disordered regions, and two conserved structural regions, with a total mass of approximately 45 kDa^26^. N protein is found in the plasma of the COVID-19 patients, while its high plasma concentrations make it a reliable biomarker of infection and disease severity. A plasma concentration of 1,000 pg/mL or higher (up to 10,000 pg/mL) is associated with a higher likelihood of severe disease manifestations^27–30^. N protein has also been implicated in triggering a cytokine storm, indicating its potential role as a target in managing severe COVID-19^31^.

In this study, we hypothesized that the N protein, which is found in high concentrations in human plasma during infection and interferes with the host immune system, might be involved in complement evasion or modulation. Thus, we aimed to explore: (1) whether the N protein interacts with the virion via spike (S) protein, (2) whether proteins N and S interact with soluble CRPs in human serum, and (3) whether binding of CRPs on the virion is mediated by N protein. Here, we report that neither the S protein nor the SARS-CoV-2 virion could bind any of the tested CRPs (C1 inhibitor (C1-INH), C4BP, fH, and VTN), on the other hand, the N protein readily bound all four CRPs. It was also found that N-CRP complex can bind virion via its interaction with the S protein. To our knowledge, this is the first report that unfolds a novel interaction between N-CRP and SARS-CoV-2 virion, which warrants further investigation.

## Results

### N protein interacts with S protein

**Fig. 1:**
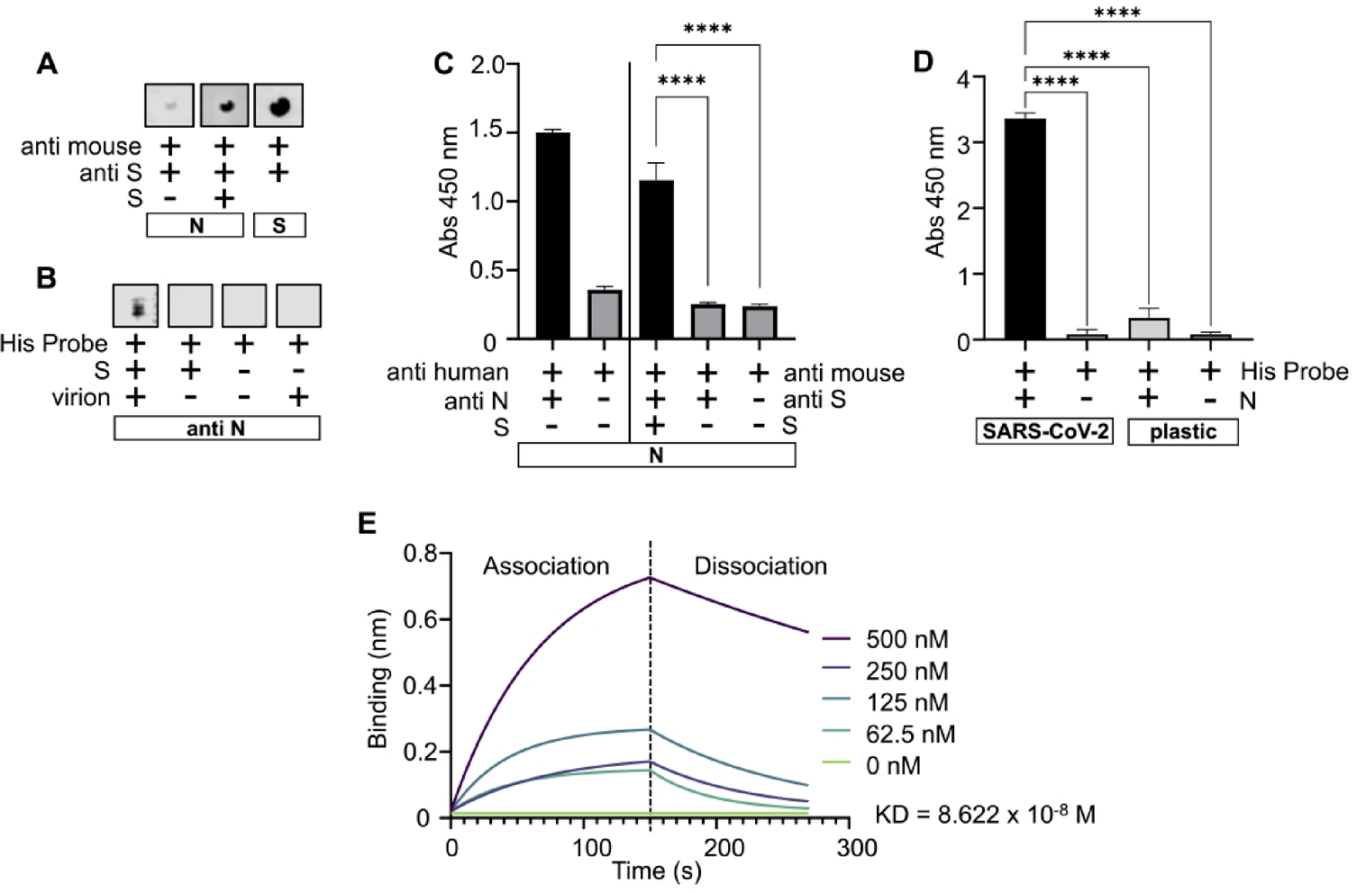
N protein interacts with S protein. Panel A – Dot blot showing interaction between S and N. Proteins immobilized on nitrocellulose membrane are in rectangles. Original blots are in Supplementary Figure S6. Panel B - Dot blot showing interaction between S and virus derived N. Anti N antibody was immobilized on nitrocellulose membrane (in rectangle. Original blots are in Supplementary Figure S7. Panel C– ELISA confirming interaction between S and N. Anti-N antibody was used to confirm coating of N protein. Coated N protein is in a rectangle. Panel D – ELISA confirming binding of N protein on SARS-CoV-2 virion fixed in the wells. Negative control – wells without coated viruses are marked as plastic. Panel E **-** Biolayer interferometry was used to assess affinity between S and N. Different concentrations of N were used in 5 runs and the result was analyzed in GraphPad Prism 9 using non-linear regression equation for association and dissociation. **** represents statistically significant difference - p value < 0.0001 (t-test).

Using a dot blot assay, the interaction between recombinant S and N proteins was detected. Specifically, 500 ng of N protein immobilized on an NC membrane formed a complex with recombinant S protein, which was detected by an S-specific antibody. Replacing the S protein with BSA in the experiment resulted in a considerable reduction in the signal (Fig. 1, Panel A). A very weak signal seen in Fig. 1, Panel A could be due to a slight non-specific reaction in the dot blot. To see whether N protein from different sources will interact with S protein, virus derived N protein was used in antibody capture dot blot. As seen in Fig. 1, Panel B the virus derived N protein captured on anti N monoclonal antibody interacted with S protein. The interaction was confirmed with an ELISA, in which the S protein bound to N, fixed in the microtiter well, was detected with S-specific antibody and TMB substrate (A_450_ = 1.15). Omitting either S protein or antibody from the assay resulted in a significant reduction in absorbance (A_450_ <0.26, *p* < 0.001; Fig. 1, Panel C). To determine the biological relevance of the S-N interaction, another round of ELISA was employed to see if an N-S complex could be formed on the surface of SARS-CoV-2 virions. As shown in Fig. 1, Panel D, the N protein readily bound to the virion, and this binding was selective, as none of the negative control reactions exhibited absorbance greater than A_450_ = 0.33 (*p* < 0.0001). Finally, the biolayer interferometry used to assess the S-N binding strength revealed that the binding affinity (KD) between two proteins was 8.622 × 10⁻⁸ M (Fig. 1, Panel E).

### Identification of plausible binding pockets on S protein for N

**Fig. 2:**
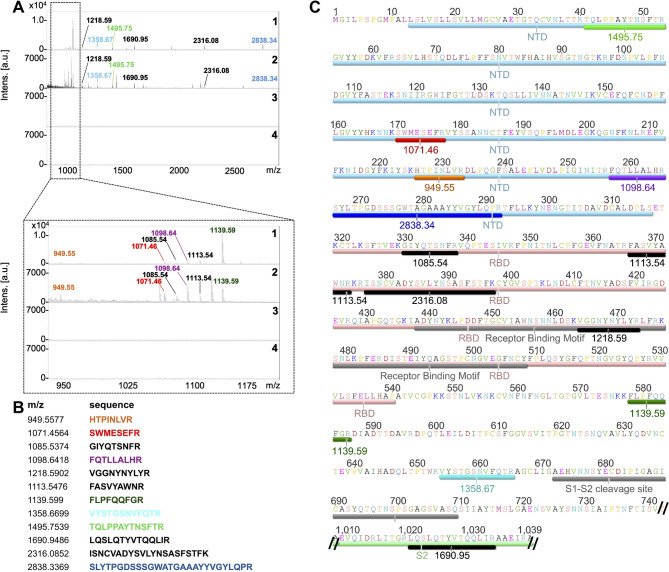
Identification of plausible binding pockets on S protein for N. Panel A – Identification of peptides of S plausibly involved in the binding with N (N was immobilized on the membrane). Identification was performed with limited tryptic digestion of protein N and S complex. **1**: MALDI-TOF spectrum of in-solution limited tryptic digestion (1 h) of S protein. **2**: N protein was immobilized on PVDF membrane, allowed to interact with S protein, the complex was trypsinized, and bound peptides of S protein to N protein were retrieved and analyzed on MALDI-TOF-MS. **3**: Negative control – S protein was omitted from the assay shown in 2. **4**: Negative control – N protein was omitted from the assay shown in 2. Panel B – List of peptides generated by in silico tryptic digestion of S protein, which were matching with peptides from in-solution digestion of S protein (Panel A.1) and on-membrane digestion of S-N complex (Panel A.2). These peptides were considered as plausible binding sites on protein S for N. Both predicted and observed (Panel A) masses are [M + H- Amino acid sequence of S protein (PDB: 6VXX) with highlighted S protein features. Peptides identified by domain mapping are highlighted in colours corresponding to the table in Panel B.

Plausible binding sites on the S protein for N were determined by employing limited proteolysis of the S-N protein complex formed on the PVDF membrane. This approach revealed 12 peptides of S that could juxtapose to N to form an S-N complex (Fig. 2, Panels A and B). The positions of these peptides on the amino acid sequence of the S protein (PDB: 6VXX) indicated that they are localized in the N-terminal domain (NTD), the receptor binding domain (RBD), and the S2 domain. Five of the peptides were localized on the NTD, four were derived from the RBD domain, two were from the region upstream of the S1-S2 cleavage site, and one (_1020_LQSLQTYVTQQLIR_1033_, 1690.94 Da) from the S2 domain (Fig. 2, Panel C). Peptide _464_VGGNYNYLYR_473_ (1218.59 Da), which was derived from RBD, is located in the central region of the receptor binding motif. The peptides identified in the mass spectrometry were derived specifically from the S-N complex, no nonspecific peptides were leached out from the PVDF membrane when either S or N was omitted from the assay (Fig. 2, Panel A).

**Fig. 3:**
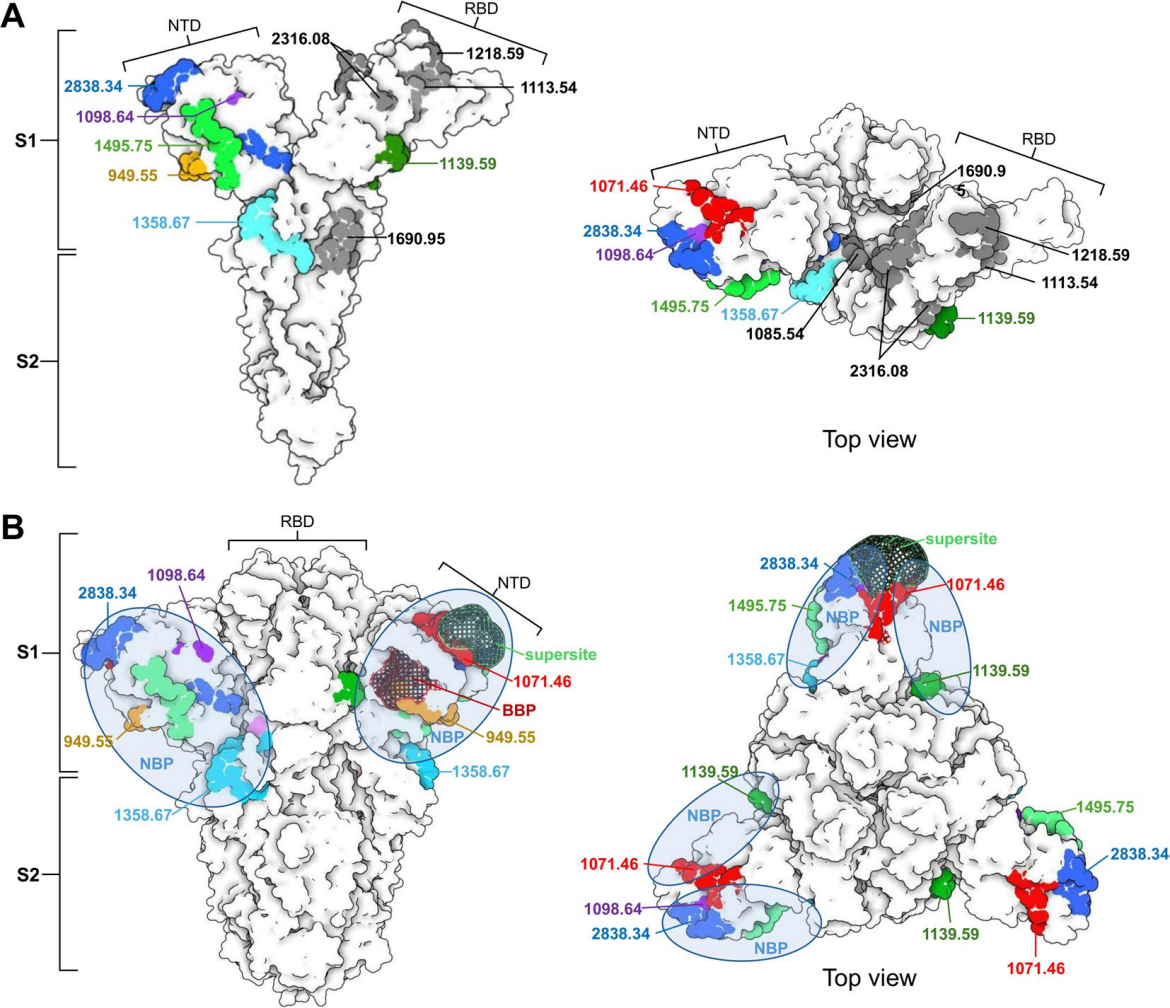
Plausible binding sites on S protein for N. Panel A – Crystal structure of S protein monomer (PDB: 6VXX) in which all identified peptides are highlighted. The colours are based on the table in Fig. 2, Panel B. A total of seven peptides were considered as plausible N-binding sites. Peptides highlighted in grey were considered not relevant because of their localization on the receptor-binding domain (RBD), or internal surface of the S protein monomer. Panel B – Plausible binding sites were highlighted on the S trimer to reveal the putative N protein binding pocket (NBP), which overlaps with the biliverdin binding pocket (BBP) and N-terminal domain (NTD) antigenic supersite.

All identified peptides were also mapped on the crystal structure (PDB: 6VXX) of S protein monomeric (Fig. 3, Panel A) and trimeric forms (Fig. 3, Panel B). Mapping indicates that peptides from NTD, could form a putative N binding pocket, however only three peptides _226_HTPINLVR_233_ (949.55 Da), _170_SWMESEFR_177_ (1071.46 Da) and _41_TQLPPAYTNSFTR_53_ (1495.75 Da) are fully surface exposed to be able to participate in the interaction. The peptide _266_SLYTPGDSSSGWATGAAAYYVGYLQPR_292_ (2838.34 Da) is partially buried with residues _266_S to S_275_ and _286_V to R_292_ being exposed on the surface. The majority of the amino acids of _257_FQTLLALHR_265_ (1098.64 Da) are buried within the NTD, with only Q_258_ and H_264_ being surface exposed.

Peptide _654_VYSTGSNVFQTR_665_ (1358.67 Da), which lies upstream of the S1–S2 cleavage site, is fully surface exposed on the S monomer. Although its position is far from the NTD in the primary sequence (Fig. 2, Panel C), the spatial folding of the protein brings it into proximity with the NTD in the secondary structure, suggesting a possible role in forming the N protein binding pocket (Fig. 3 Panel A). Another peptide upstream of the S1–S2 cleavage site, the _578_FLPFQQFGR_586_ (1139.59 Da), is fully surface exposed on monomeric S protein, however in trimeric form, it seems largely buried with just _578_F to Q_582_ residues surface-accessible (Fig. 3, Panel B).

Peptides _1020_LQSLQTYVTQQLIR_1033_ (1690.94 Da) and _330_GIYQTSNFR_338_ (1085.54 Da) are exposed to the internal surfaces of S2 domain and RBD domain, respectively, making them inaccessible for N protein in the S protein trimer (Fig. 3, Panel A).

### N protein interacts with soluble CRPs

**Fig. 4:**
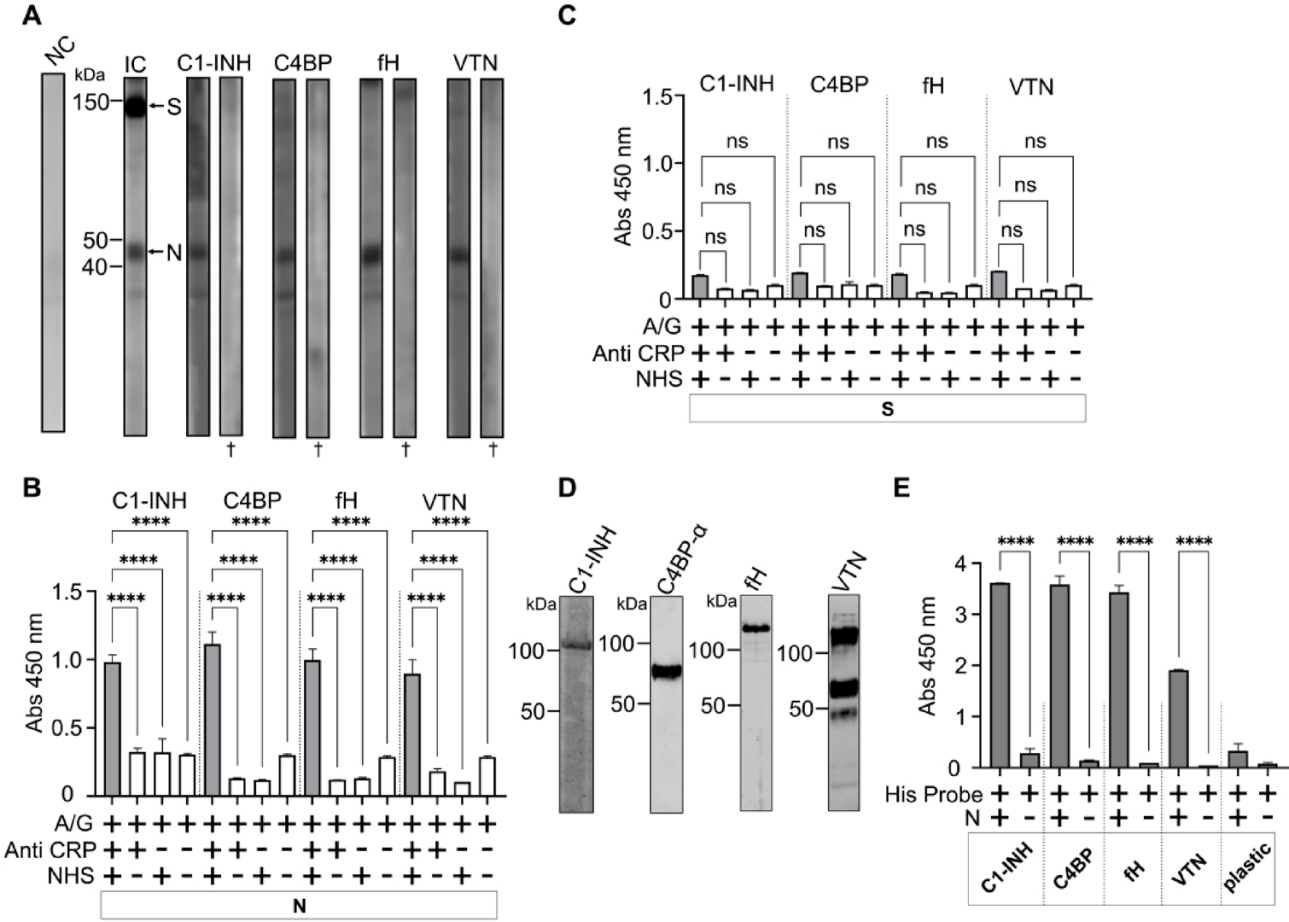
N protein interacts with soluble CRPs. Panel A – Far Western blots showing interaction of S and N proteins and soluble CRPs. Input control (IC) shows that both S and N proteins had expected masses of ~ 150 kDa and ~ 48 kDa, respectively. N protein interacts with all soluble CRPs from serum. NC - negative control in which anti-CRPs antibodies were omitted from the assay. † indicates denatured proteins on membrane. Original blots are in Supplementary Figures S8 – S10. Panel B – Binding of CRPs by N assessed by ELISA. 500 ng of N (in rectangle) were coated on the wells of the ELISA plate and incubated with diluted normal human serum (NHS). Interactions were detected using CRP-specific antibody and HRP-conjugated protein A/G. N interacted with C1 inhibitor (C1-INH), C4BP, Factor H (fH) and Vitronectin (VTN). Omitted NHS and primary antibody from the assay served as negative controls. Panel C – Assay was performed exactly as in Panel B, however S protein was immobilized in the wells. Panel D – Purification of CRPs. Western blots confirming the presence of CRPs detected by CRP-specific antibodies. CRPs had expected molecular masses (fH ~ 150 kDa, C1-INH ~ 105 kDa, VTN ~ 70 kDa and C4BP-alpha chain ~ 70 kDa). Full**-**length Western blots are available in - The interactions between N and soluble CRPs purified from NHS were confirmed using ELISA. The CRPs were coated in the wells (200 ng/well) and incubated with N. Corresponding coated CRPs are in rectangles. Omitting N and coating of CRPs (plastic) served as negative controls. Abs 450 nm– Absorbance at 450 nm, **** represents statistically significant interactions with p value < 0.0001 (t-test), ns – not statistically significant.

Far Western blot was used to determine whether S and N proteins interact with soluble CRPs, namely C1-INH, C4BP, fH, and VTN from normal human serum (NHS). Figure 4, Panel A shows that N protein interacts with all four soluble CRPs, whereas S protein did not show any interaction. It should be noted that the S and N proteins used in far-WB were renatured on-membrane. Heat-denaturated S and N transferred on membrane showed no interaction with CRPs (Fig. 4, Panel A), implying that protein structure is important for interactions. An ELISA-based approach was used to confirm the result of far Western blot. N protein incubated with the NHS formed complexes with CRPs, which were detected using specific antibodies (A_450_ > 0.8, *p* < 0.0001, Fig. 4, Panel B), however the S protein displayed no significant interaction (A_450_ < 0.2, Fig. 4, Panel C). To further validate the interaction, CRPs purified from the serum were used in ELISA assay. While C1-IHN and fH were procured commercially, C4BP and VTN were purified in this study using antibody-affinity chromatography. After validating the presence of purified CRPs with Western blot analysis (Fig. 4, Panel D), they were coated in the wells and allowed to interact with N in an ELISA. The results reveal that N protein forms complexes with each of the purified CRPs examined here, and the reaction was specific (A_450_ > 1.9, *p* < 0.0001, Fig. 4, Panel E), since all control groups, i.e. either N, CRPs, or both excluded from the assay, did not show any significant interaction.

The biolayer interferometry used earlier to assess the S-N binding strength could be employed to reveal binding affinities between N or S protein binding to CRPs, which gives more reliable results than WB and ELISA, as it is a label-free concept. In this study we could not perform biolayer interferometry especially in the case of N-CRPs, as biotinylation of the N resulted in precipitation.

### N protein can recruit CRPs on the virion

**Fig. 5:**
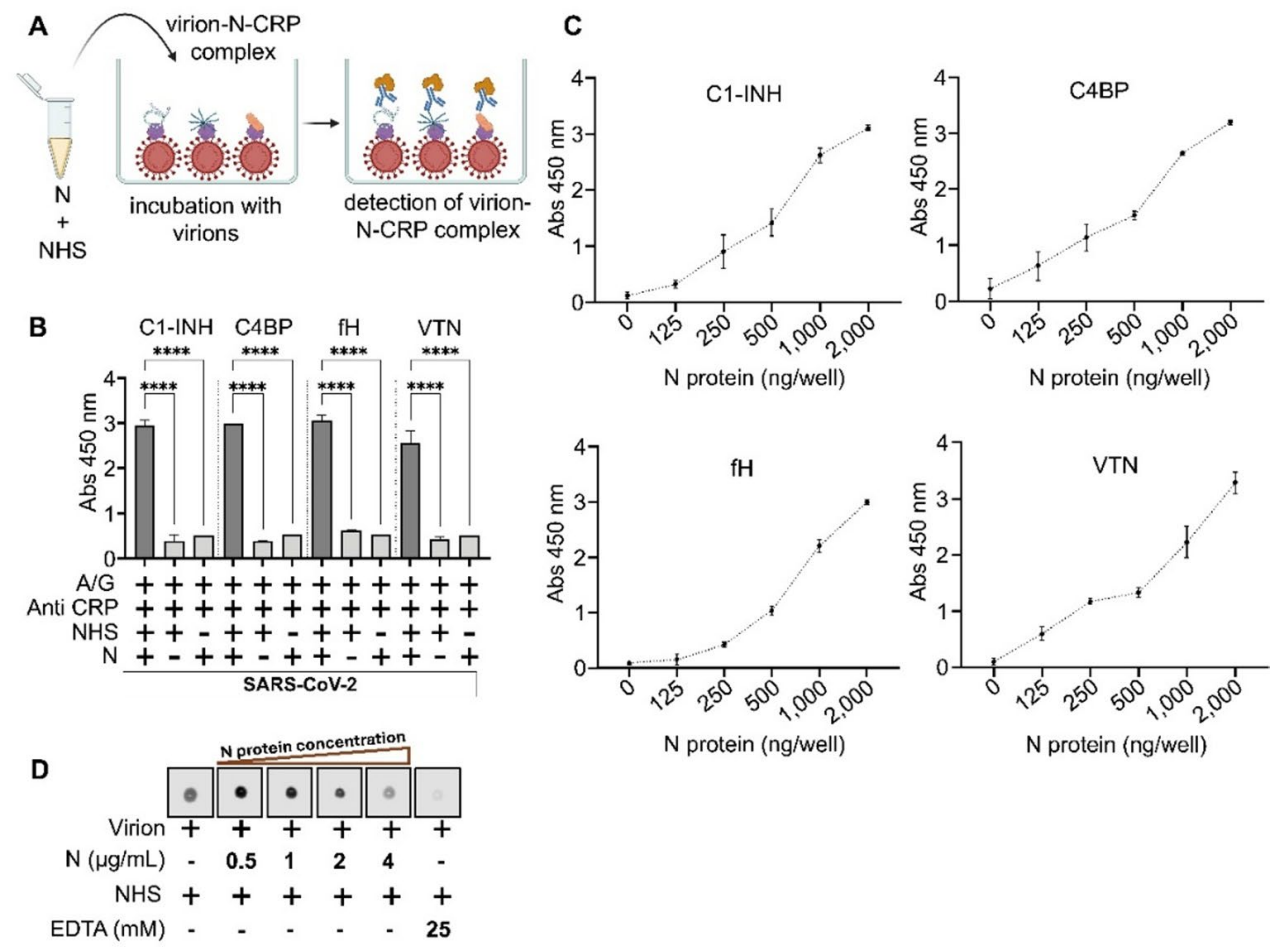
N protein recruits soluble CRPs on the surface of virion. Panel A – Method overview. N was pre-incubated with NHS for 1 h prior to incubation with inactivated SARS-CoV-2 (virions present in concentrated cell culture medium). The tripartite complex of virion-N-CRP was detected using CRP-specific antibodies and HRP-conjugated protein A/G. Created in BioRender. Viglasky, J. (2025) https://BioRender.com/f83l435 Panel B – Recruitment of CRPs by N protein was assessed by ELISA. Virions in the wells were incubated with a mixture of N and NHS, and CRPs were detected on SARS-CoV-2 virions using CRP-specific antibodies. CRPs bound to virions only when N was present in the NHS. Omitting NHS from the assay served as negative controls. Panel C – Dose dependent recruitment of soluble CRPs by N protein. The assay was performed as in Panel B, however, 2-fold dilutions of N protein were mixed with NHS and incubated with virions. The result shows that recruitment of CRPs on the virion by N is dose dependent. **** represents statistically significant interactions with p value < 0.0001 (t-test). Panel D – Dot blot result showing C5b-9 deposition on virions (formation of terminal complement complex on virion) when incubated with NHS (first dot from left hand side). Preincubation of the N protein with NHS, prior to incubation with virion caused decreased in the formation of terminal complex. The effect was N protein concentration dependent (0.5 to 4 µg/mL). As a control, NHS was supplemented with EDTA, which blocks activation of the complement cascades. Full scan of membrane is in Supplementary figure S12.

When preincubated N protein in NHS (1 h, room temperature) was used in ELISA, in which inactivated SARS-CoV-2 Omicron virions (present in concentrated cell culture medium), were fixed in the wells, we observed that N protein might have formed a complex with CRPs during preincubation, and the whole complex could bind to the virion (Fig. 5, Panels A and B). In other words, it is tempting to believe that N protein facilitates the recruitment of CRPs onto the virus. When NHS was omitted from the assay, no interaction was observed. When the N was omitted from assay, none of the CRPs from NHS could bind to the virion (Fig. 5, Panel B). Thus, we presumed that CRP binding to virion should be dependent on the concentration of N protein. A positive correlation between the binding CRPs on virion and N concentration was found when N protein at varying concentrations (2000 ng to 125 ng/well) was preincubated with NHS and subsequently used in ELISA (Fig. 5, Panel C). It is important to note that in ELISA employed to assess recruitment of CRPs onto the virus, mediated by protein N (Fig. 5, Panel B), concentrated cell culture medium containing virions was used, which comprises additional components. Components from the medium can give non-specific signals.

### N protein decreases the MAC deposition in virion

The effect of N protein on complement activation was determined by detecting the formation of the C5b-9 terminal complex. First, when virions (purified with sucrose gradient) were incubated in NHS, we observed the formation of the MAC complex, as the deposition of C5b-9 was detected. Preincubating NHS with different concentrations of N protein (0.5 to 4 µg/mL) and then incubating with virions resulted in decreased deposition of C5b-9 (Fig. 5, Panel D). As a control, we excluded N protein from the experiment, wherein the C5b-9 deposition was evident. Another control, in which the complement was inactivated by adding EDTA, revealed no obvious formation of the terminal complex.

## Discussion

N protein is present not only in the cytosol of SARS-CoV-2 infected cells, but also on the cell surface and in plasma^27–30,32^. It has been identified as VIP, which binds to at least 11 human chemokines and inhibits chemotaxis^32^. N protein increases proinflammatory cytokines (IL-6, IL-12, IL-1β, and TNF-α) and modulates genes associated to apoptosis (BAK, BAX, and BCL-2)^33^. As several studies found high levels of N protein in the plasma of COVID-19 patients and demonstrated its immunomodulatory potential^34–37^, we hypothesized that N protein could interact with the proteins of the complement cascade, especially CRPs. Several viruses, primarily from the *Flaviviridae*, *Retroviridae*, and *Togaviridae* families, use secreted viral proteins to prevent complement system activation by recruiting soluble CRPs (reviewed in^38^). Some studies have proposed a theory of depletion of complement activators by releasing a large amount of secreted proteins (like NS1 by Flaviviruses) that attract CRPs, some have shown binding of CRPs directly on virions (HIV binds fH on its surface via gp41 and gp120^39^), some have demonstrated binding of secreted protein-CRP complex on infected cells to protect them from complement activation (e.g., NS1-C4BP on infected cells^14^ or formation of MAC (e.g. NS1-VTN^16^. Based on the fact that NS1 readily binds to the E protein of some of the members of *Flaviviridae*^40^, complement evasion by NS1-CRP complex binding to the virion has been proposed. To this background, it was intriguing in our investigation to evaluate whether the N protein has an affinity for S protein and SARS-CoV-2 virion, and whether it can recruit soluble CRPs. Results from our study, as well as those of others^41^ confirm the interaction between N and S proteins of SARS-CoV-2 (Fig. 1). The measured KD of S-N interaction is in the upper nanomolar range, making it as strong as typical antibodies, which usually have low micromolar to picomolar affinities^42^. The strong affinity between N and S proteins suggests that a low concentration of N protein might be sufficient for binding to S protein on virions in plasma. We further presume that S-N interaction has biological relevance, since our data reveals that the interaction is not specific to recombinant forms of the proteins but can also occur between N and the virion.

Mapping of the plausible binding sites for N on the S protein (Figs. 2 and 3), revealed that interaction between S and N might be through the NTD of S protein as five of all identified peptides were located on this region and two were from its proximity in the secondary structure (1358.67 Da and 1139.59 Da, Fig. 3). NTD of S protein is one of the dominant targets for neutralizing antibodies and contains binding sites for various host molecules such as heme metabolites and sialic acid^43–45^. A total of six antigenic sites (i, ii, iii, iv, v and vi) are located on NTD, with site i (supersite, shown in Fig. 3 panel B) being the target for most NTD-specific neutralizing mAbs^45^. Apart from containing important epitopes, the NTD also binds biliverdin to so-called biliverdin binding pocket (BPP, shown in Fig. 3, Panel B). Binding of biliverdin significantly reduces SARS-CoV-2 susceptibility to neutralizing antibodies^44^. This reveals that NTD has the potential to interact with diverse molecules, and binding of N protein via NTD is highly plausible. Peptides constituting putative N binding pocket (_41_TQLPPAYTNSFTR_53_, _170_SWMESEFR_177_, _226_HTPINLVR_233_, _578_FLPFQQFGR_586_ and _266_SLYTPGDSSSGWATGAAAYYVGYLQPR_292_) identified in this study are overlapping with supersite and BBP (Fig. 3, Panel B).

Four identified peptides (_330_GIYQTSNFR_338_, _366_FASVYAWNR_374_, _464_VGGNYNYLYR_473_, and _377_ISNCVADYSVLYNSASFSTFK_397_) overlapped with RBD (highlighted in grey in Fig. 3 panel A). Of these four peptides, the _330_GIYQTSNFR_338_ is on the internal surface of RBD, making it inaccessible in the S protein trimer. Likewise, the _1020_LQSLQTYVTQQLIR_1033_ peptide (1139.59 Da) is mapped on the internal surface of the S2 domain (Fig. 3 panel A). Binding of N to RBD would interfere with essential functions of S protein such as receptor recognition and binding^46^. Thus, these five peptides were not considered as plausible N binding sites. It is possible that those five peptides were produced during limited tryptic digestion of the N-S complex, as the experiment used monomeric form of recombinant N and S, which may have allowed N to bind to otherwise inaccessible site of S. Based on our data showing that N protein can simultaneously binds to SARS-CoV-2 virion as well as CRPs (Fig. 5, Panel B), we can hypothesize that the binding sites of CRPs and S protein on N do not overlap, allowing the complex of virion-N-CRP to be formed.

This study clearly demonstrates that N protein interacts with soluble CRPs from human serum (C1-INH, C4BP, fH, and VTN), however, neither S protein (recombinant form) nor virion can bind any of these regulators. In contrast to our findings, an interaction between SARS-CoV-2 virions and fH has previously been reported^21^; however, it is important to note that this interaction was between fH and an envelope-membrane fusion protein, rather than recombinant S protein. A similar contradiction was also seen in an earlier report, which states the interaction between C4BP and SARS-CoV-2 pseudovirus particles^47^.

Interactions of SARS-CoV and SARS-CoV-2 encoded proteins with C1-INH have been reported with the suggestion that inhibition of C1-INH by CoV proteins might lead to excessive activation of the complement cascade and intrinsic coagulation cascade^48^. C1-INH is the multifunctional glycoprotein that inhibits the classical pathway by preventing the formation of C1 complex (i.e. via inhibition of C1r and C1s). C1-INH also inhibits the lectin pathway of complement activation via interaction with mannan-binding lectin-associated serine protease-1 and − 2 (MASP1 and MASP2) and inhibits the alternative pathway by binding to C3b^49^. The N protein binding to C1-INH may deplete the free C1-INH in serum, leading to excessive activation/dysregulation of the complement cascade. Furthermore, recruitment of C1-INH to the virion surface, mediated by N-S interaction, may lead to an increase in the concentration of C1-INH on the virion. This hypothesis, however, merits experimental validation in the future. A similar assumption could be made in the case of C4BP and fH binding to N protein. It has earlier been shown that the binding of flaviviral NS1 protein to C4BP modulates complement activation in the fluid phase or on the surface of infected cells^14^. Binding of C4BP on the cell surface mediated by NS1 can block activation of the classical pathway as well as lectin pathway^50^. Binding of fH on the pathogen surface, on the other hand, ensures inhibition of the alternative pathway. Factor H is the major regulator of the complement system, inhibiting its activation by binding to C3b and accelerating the decay of alternative C3 convertase. It also possesses a cofactor activity for factor I-mediated inactivation of C3b^51^. SARS-CoV-2 is known to compete with fH on the surface of host cells, leading to complement dysregulation on the cell surface^52^.

Complement evasion strategies employed by pathogens are intricate, and it is even more intriguing to understand how the same pathogen can exploit multiple CRPs to evade complement cascades as they progress through the activation. Viruses and bacteria can increase the concentration of VTN on their surfaces, which binds to the C5b-7 complex and destabilizes the formation of the membrane attack complex^53,54^. The NS1 protein of the dengue virus is known to inhibit C9 polymerization by binding to VTN^16^. It is thus tempting to explore in future if the similar mechanisms exist for SARS-CoV-2 mediated by N protein.

We evaluated if the MAC is formed on the virion and that the development of MAC is attenuated by protein N in a dose-dependent manner. As the activation of complement leads to the formation of C5b-9 terminal complex, which ultimately cause the lysis (cell lysis or bacteriolysis or virolysis), detection of the deposition of C5b-9 on to the virion serves as a good marker to determine complement evasion. Our findings suggest that N protein may reduce the deposition of C5b-9 on the virion surface (Fig. 5, Panel D). These findings merit further investigation using various techniques such as viral neutralisation test in the presence/absence of protein N.

In summary, this study suggests a plausible role of the SARS-CoV-2 N protein in complement modulation, extending previous insights into its diverse immunomodulatory functions. We demonstrate that the N protein not only exhibits a strong affinity for the S protein and virion surface but also interacts with soluble CRPs. These findings suggest that N protein may act as a molecular bridge facilitating the recruitment of complement inhibitors to the virion, thereby attenuating complement activation and MAC formation. Such a mechanism parallels strategies employed by other viruses and their proteins, such as the flaviviral NS1, and suggests an additional layer of SARS-CoV-2 immune evasion. Further investigation is warranted to delineate the structural determinants and stoichiometry of N–CRPs interactions, as well as their functional impact on complement activation in physiological and pathological contexts. To validate the suggested paradigm, molecular interfaces between N–CRPs will need to be elucidated using suitable molecular techniques along with their role in complement evasion in in vitro and in vivo setups. Finally, addressing N-CRPs interactions may offer a promising therapeutic target for restoring complement-mediated antiviral defense or mitigating excessive complement activation seen in severe COVID-19.

## Methods

### Viral proteins and antibodies

The cloning, expression, purification, and quality control of recombinant SARS-CoV-2 spike protein are described in detail in Supplementary information 1. All antibodies and proteins used are listed in Supplementary information 2.

### Propagation of virus and Titration

The strain Slovakia/SK-BMC-BA42/2022 was isolated in July 2022 (https://www.european-virus-archive.com/virus/sars-cov-2-strain-slovakiask-bmc-ba422022-omicron-voc-ba52) and belongs to the BA.5.2 lineage (Omicron VOC). The identity of the viruses was confirmed by sequencing. The complete genome sequences are available in the EVAg portal.

Vero E6 cells were cultivated in DMEM (Sigma-Aldrich, USA) medium supplemented with 5% fetal bovine serum (Biowest, France), 2 mM L-glutamine (Serana, Germany), 50 U of penicillin and 50 µg/mL of streptomycin (Jena Bioscience, Germany). At 80% confluency, cells were infected at MOI 10 and incubated for 48 h at 37 °C in 5% CO_2_. After incubation, the flask containing infected cells was subjected to a freeze/thaw cycle twice, and the whole content was retrieved and centrifuged at 2790 x g for 10 min to remove cellular debris. Supernatant was concentrated by ultrafiltration spin column (VivaSpin, 100 kDa MWCO, Cytiva, USA) as per manufacturer’s instructions. The virus titer in concentrated supernatant was determined using plaque assay (details presented in Supplementary information 3).

### Virus ultracentrifugation and purificaiton

Vero E6 cells cultivation and SARS-CoV-2 infection were carried out as described in previous section. Flasks containing infected cells were subjected to a freeze/thaw cycle twice, and the whole content was retrieved and centrifuged at 2790 x g for 10 min to remove cellular debris. Second centrifugation was carried out at 12 000 x g for 10 min. Precleared supernatant was placed on 20%−30% sucrose gradient (prepared in PBS, pH 7.3) and centrifuged on Himac CS150NX micro-ultracentrifuge, rotor S50A (Himac, Japan) at 100 000 x g, 4 °C for 22 h. After centrifugation, supernatant was carefully removed, and viral pellet was resuspended in sterile PBS (pH 7.3). Total of 4 T75 flasks of SARS-CoV-2 infected cells were processed amounting to 1.5 mL of purified virus in PBS. Purified virus was directly used for further experiments.

### Assessment of interaction between S and N proteins

Dot blot was used to assess interaction between S and N proteins. First, 500 ng of N protein was spotted on nitrocellulose membranes and left to dry. The membranes were blocked with 5% BSA in TBS (50 mM Tris and 150 mM sodium chloride, pH 7.3) for 1 h at room temperature and washed once with TBST-20 (TBS with 0.05% Tween 20). The membrane was then incubated with 1 mL of S protein (3 µg/mL in 1% BSA in TBST-20) for 1 h with gentle shaking at room temperature. After incubation, the membrane was washed with TBST-20 three times and then incubated for 1 h with SARS-CoV-2 spike protein (RBD) antibody diluted 1:2,000 (v/v) in 0.5% BSA in TBST-20. The membrane was washed three times with TBST-20 and incubated for 1 h with IRDye 800CW donkey anti-mouse IgG (H + L) antibody diluted 1:15,000 (v/v) in 0.5% BSA in TBST-20. The membrane was washed three times with TBST-20 and scanned on Odyssey CLx instrument at 800 nm (LiCor, USA). As a negative control, incubation with S protein was omitted from the assay. S protein immobilized on the membrane and detected with primary SARS-CoV-2 spike protein (RBD) antibody and secondary IRDye 800CW donkey anti-mouse IgG (H + L) antibody served as an input control.

### Interaction of virus-derived N protein and S protein

Antibody capture dot blot was used to detect interaction between virus-derived N protein and recombinant S protein. First, ultracentrifuged and heat-inactivated virions were sonicated for 10 s to disrupt viral membrane. Then, 1 µg of anti-N protein monoclonal antibody was spotted on nitrocellulose membranes and left to dry. The membranes were blocked with 5% BSA in TBS for 1 h at room temperature and washed once with TBST-20. The membranes were then incubated with sonicated virions containing virus-derived N protein. After capture, membranes were washed with TBST-20 three times and then incubated with 1 mL of His tagged S protein (5 µg/mL in 0.5% BSA in TBST-20) for 2 h with gentle shaking at room temperature. Membranes were washed three times with TBST-20 and incubated with HRP-conjugated His-probe diluted 1:5,000 (v/v) in 0.5% BSA in PBST-20 for 1 h at room temperature. Final washing was done three times with TBST-20 and one time with TBS. The interactions were detected with SuperSignal West Dura (Thermo Fisher Scientific) and chemiluminescence signals were captured on C-Digit blot scanner (LiCor). For negative control, either incubation with virion or S protein or both were omitted from assay.

### Assessment of interaction between S and N proteins with ELISA

500 ng of N protein resuspended in 100 µl of coating buffer (100 mM sodium carbonate, pH 9.5) was coated in a 96-well plate overnight at 4 °C (in triplicate). The wells were blocked with 5% BSA in PBS (137 mM NaCl, 8 mM NaH_2_PO_4_, 2mM KH_2_PO_4_, 2.7 mM KCl; pH 7.4) for 1 h at room temperature, and 100 µL of S protein (3 µg/mL resuspended in PBS) was added in the coated wells. After 1 h of incubation with constant shaking (350 RPM) at room temperature, the wells were washed three times with PBST-20 and incubated with primary SARS-CoV-2 spike protein (RBD) antibody diluted 1:500 (v/v) in 0.5% BSA for 1 h with constant shaking (350 RPM). Wells were washed three times with PBST-20 and incubated with anti-mouse IgG goat polyclonal antibody (HRP) diluted 1:20,000 (v/v) in 0.5% BSA for 1 h with constant shaking (350 RPM) at room temperature. Wells were washed three times with PBST-20 and once with PBS. Ultra-TMB ELISA substrate (Thermo Fisher Scientific, USA) was added (100 µL) for 25 min, and the reaction was stopped with 2 M H_2_SO_4_. Absorbance was measured at 450 nm (BioTek 800 TS, Agilent, USA). For negative control, either S protein or both the primary antibody and S protein were omitted from the reaction. All controls were performed in triplicate. To confirm coating of N protein, control wells were incubated with SARS-CoV-2 nucleocapsid protein human monoclonal antibody (diluted 1:500 (v/v) in 0.5% BSA) for 1 h with constant shaking (350 RPM). The wells were washed three times with PBST-20 and incubated with anti-human IgG (HRP) diluted 1:20,000 (v/v) in 0.5% BSA for 1 h with constant shaking (350 RPM) at room temperature. After washing, Ultra-TMB was used for colorimetric analysis as described above.

### Binding of N protein on the SARS-CoV-2 virion

First, the binding of N protein on the virion was tested using ELISA. In short, 10 µL of medium from the infected cells, concentrated by ultrafiltration spin column, containing 1 × 10^9^ PFU was deposited in the center of each well (96-well ELISA plate), allowed to dry, and then 100 µL of 4% paraformaldehyde was added and incubated for 10 min. Wells were washed with water once, and the plate was placed upside down in 100% methanol for 5 min to disinfect the plate thoroughly to be able to transport it out of BSL-3 biocontainment room. The wells were washed with PBST-20 and blocked with 5% BSA in PBS for 1 h at room temperature. After blocking, 100 µL of N protein (1 µg/mL in 0.5% BSA in PBST-20) was added to each well and incubated for 1 h at room temperature. Wells were washed three times with PBST-20 and incubated with HRP-conjugated His-probe diluted 1:5,000 (v/v) in 0.5% BSA in PBST-20 for 1 h at room temperature. Final washing was done three times with PBST-20 and one time with PBS. After the final washing, Ultra-TMB ELISA substrate was added for 25 min, and the reaction was stopped with 2M H_2_SO_4_. Absorbance was measured at 450 nm. For negative control, either N protein or virus was omitted from the assay.

### Biolayer interferometry for assessment of binding affinity between protein S and N

The affinity of interaction between S and N proteins was measured using the Fortebio BLItz system (Fortebio, USA). First, the biotinylated S protein (15 µg/mL) was loaded on Octet SAX2 streptavidin biosensors (Fortebio) for 120 s with a shaker speed of 2200 RPM, followed by washing for 120 s with a shaker speed of 2200 RPM. PBST-20 was used as a sample buffer and kinetics buffer. The run settings were: 30 s for baseline, followed by association for 120 s and dissociation for 120 s. All steps were carried out at a shaker speed of 2200 RPM. All N protein samples were run in concentrations of 62.5 nM, 125 nM, 250 nM and 500 nM. PBST-20 without N protein was used as a negative control. Data was analyzed in GraphPad Prism 9.5.1 for Windows (GraphPad Software, USA, www.graphpad.com) using a nonlinear regression equation for association and dissociation.

### Mapping of the plausible binding sites of N protein on S protein

Mapping of the binding sites was performed as per the protocol published previously^55^. Briefly, 2 µg of N protein was spotted on an activated Immobilon-FL PVDF membrane (Millipore, USA) and air-dried overnight. The membrane was submerged in TBS containing 4 µg of S protein for 2 h at room temperature with gentle shaking. The membrane was washed six times with TBS for 1 min and dried overnight prior to trypsin digestion. Limited trypsin digestion of the N-S complex was performed with 1 µg of Trypsin Gold (Promega, USA) diluted in 300 µl of pre-warmed (37 °C) ammonium bicarbonate buffer (20 mM, pH 7.6) for 1 h. The membrane was washed 6 times with ammonium bicarbonate buffer. To elute peptides interacting with N protein, the membrane was vortexed for 1 min in 10 µl of ~ 98% formic acid (Sigma-Aldrich), followed by adding 50 µl of ≥ 99.9% acetonitrile (Sigma-Aldrich). The membrane was vortexed again, and supernatant was retrieved. The sample was vacuum-dried at 60 °C, and the pellet resuspended in 5 µl of TA50 (1:1 [v/v] acetonitrile: 0.1% trifluoroacetic acid in water, Sigma-Aldrich). After resuspension, 25 µl of 0.1% trifluoroacetic acid was added. Peptides were concentrated with ZipTip C18 (Millipore) following the manufacturer’s instructions. Peptides were eluted in α-Cyano-4-hydroxycinnamic acid (HCCA; Bruker-Daltonics, Germany) matrix dissolved in TA50. Two microliters of the eluate were spotted on AnchorChip (Bruker-Daltonics) and air-dried. The spectra were analyzed in a MALDI-TOF Microflex-LRF mass spectrometer (Bruker-Daltonics). Peptide calibration standard II (Bruker-Daltonics) was used as a calibrant. Spectra were acquired in reflectron-positive mode at a laser frequency of 35 Hz (200 shots). For negative controls, either the N protein or S protein was excluded from the protocol. Peptide masses acquired from mass spectrometry were correlated with the masses calculated from in silico trypsin digestion of S protein using PeptideMass server (https://web.expasy.org/peptide_mass/). The sequence of S protein used in in silico analysis was YP_009724390.1 (NCBI reference sequence).

### Assessment of the binding of complement regulatory proteins with S or N proteins

Interactions between recombinant S and N proteins and soluble CRPs were determined using far Western blot. First, the sample was prepared by mixing S and N protein (25 µg of each) with NuPage LDS sample buffer (Invitrogen, USA), without heating. The mixture was loaded onto a 12% Bis-Tris polyacrylamide gel (50 mm well width). Proteins were resolved in SDS–MOPS running buffer (50 mM MOPS, 50 mM Tris, 0.1% w/v SDS, 1 mM EDTA, pH 7.7) at 150 V for 2 h. After electrophoresis, the proteins were transferred to an Immobilon-FL PVDF membrane (Millipore) using a bicine/Bis-Tris (25 mM each) transfer buffer containing 20% (v/v) methanol at 30 V for 1 h. The membrane was then cut into ~ 2 mm strips, each holding ~ 1 µg of both recombinant proteins. Proteins were renatured on the membrane as described elsewhere^56^. Briefly, membranes were serially incubated in AC buffers (100 mM NaCl, 20 mM Tris (pH 7.6), 10% glycerol, 0.1% Tween-20, 2% BSA) containing 6 M, 3 M, 1 M and 0.1 M guanidine-HCl for 30 min each, with final incubation in AC buffer without guanidine-HCl at 4 °C overnight. All membranes were washed three times with TBST-20 and blocked with 5% BSA in TBS for 1 h with gentle shaking. Membranes were then washed and incubated for 2 h in NHS (diluted 1:8 (v/v) in TBST-20) collected from the pre-COVID-19 era (stored at −80 °C) to avoid false-positive results from the presence of antibodies against S and N proteins. Membranes were washed 10 times in TBST-20 and incubated with anti-CRP antibody (anti-C1 inhibitor polyclonal or anti-C4BP or anti-Factor H or anti-vitronectin) diluted 1:500 (v/v) in 0.5% BSA in TBST-20 for 1 h. Membranes were washed 10 times with TBST-20 and incubated with HRP-conjugated protein A/G diluted 1:30,000 (v/v) in 0.5% BSA in TBST-20 for 1 h. After final washing with TBST-20, the interactions were detected with SuperSignal West Dura and chemiluminescence signals were captured on C-Digit blot scanner. For negative controls, the assay was followed, omitting primary antibodies (anti-CRP antibodies) and omitting on-membrane renaturation protocol. For input control, a strip containing S and N was incubated in serum from a post-COVID-19 individual (1:200, v/v), followed by HRP-conjugated protein A/G and SuperSignal West Dura as described above.

### Confirmation of the interactions between CRPs and N protein

500 ng of N protein was coated in a 96 well plate overnight at 4 °C. The plate was washed with PBST-20 and blocked with 5% BSA in PBS for 1 h at room temperature. After blocking, 100 µL of NHS diluted 1:500 (v/v) in PBST-20 was added to the wells coated with N and S proteins and incubated for 1 h at room temperature. Incubation with 0.5% BSA in PBST-20 instead of NHS was used as a negative control. Wells were washed three times with PBST-20 and incubated with anti-CRP antibodies (anti-C1 inhibitor polyclonal or anti-C4BP or anti-factor H antibody or anti-vitronectin) diluted 1:500 (v/v) in 1% BSA for 1 h with constant shaking (350 RPM). Wells were washed three times with PBST-20 and incubated with HRP-conjugated protein A/G diluted 1:20,000 (v/v) in 0.5% BSA for 1 h with constant shaking (350 RPM) at room temperature. Final washing was performed three times with PBST-20 and one time with PBS. Ultra-TMB ELISA substrate was added for 25 min, and the reaction was stopped with 2 M H_2_SO_4_. Absorbance was measured at 450 nm. As negative controls, the S protein, or primary antibody (anti-CRP antibody) or both were omitted from the assay.

To confirm the possible interaction between S protein and CRPs, the assay was performed exactly as above, with coated S protein.

### Purification of CRPs (VTN and C4BP) from serum

200 µL of protein-capturing magnetic beads (MB-CoVAC-Select kit, Bruker-Daltonics) were incubated with 200 µg of either anti-C4BP polyclonal antibody or anti-vitronectin antibody as per the manufacturer’s instructions. After 1 h of incubation, the unbound antibodies were washed with washing buffer provided in the kit and non-specific binding sites on the beads were blocked by incubating for 2 h in freshly prepared 8% ethanolamine solution (pH 8.5). Beads were washed three times with washing buffer provided in the kit. Antibody-loaded beads were incubated in NHS diluted 1:100 (v/v) in binding buffer (provided in the kit) supplemented with 0.5% L-lysine for 1 h at room temperature. After binding, the beads were washed using a washing buffer (provided in the kit) and eluted using 50 µL of glycine-HCl (100 mM, pH 3). The pH was adjusted by adding 2.5 µL of Tris-HCl solution (20 mM, pH 9) to eluted C4BP and VTN. To confirm the presence of CRPs, 5 µL of eluate was mixed with LDS sample buffer and resolved on the 12% SDS-PAGE gel and transferred to nitrocellulose membrane as described above. The membrane was washed with TBST-20 and blocked with 5% BSA in TBS for 1 h at room temperature. The membrane was then washed with TBST-20 for 5 min and incubated with either anti-C4BP or anti-vitronectin antibodies (diluted 1:5,000 (v/v) in 0.5% BSA in TBST-20) for 1 h at room temperature. The membrane was washed three times with TBST-20 and incubated with IRDye 800CW donkey anti-rabbit IgG (H + L) antibody diluted 1:25,000 (v/v) in 0.5% BSA in TBST-20 for 1 h at room temperature. After incubation with the secondary antibody, the membrane was washed three times with TBST-20 and one time with TBS and scanned using Odyssey CLx instrument at 800 nm. CRPs procured commercially (C1-INH and fH, Supplementary information 2) were also included in the Western blot and were detected with either anti-C1 inhibitor polyclonal antibody or anti-factor H antibody, respectively.

Concentration of eluted C4BP and VTN was measured with the Bradford colorimetric assay.

### Interactions between N protein and purified CRPs

200 ng of each CRP was added in the well (96-well ELISA plate), allowed to air dry, and then fixed with 4% paraformaldehyde. The wells were washed with PBST-20 and blocked with 5% BSA in PBS for 1 h at room temperature. After blocking, 100 µL of N protein (1 µg/mL in 0.5% BSA in PBST-20) was added to each well coated with CRPs and incubated for 1 h at room temperature. Wells were washed three times with PBST-20 and incubated with HRP-conjugated His-probe diluted 1:5,000 (v/v) in 0.5% BSA in PBST-20 for 1 h at room temperature. Final washing was done three times with PBST-20 and one time with PBS. After the final washing, Ultra-TMB ELISA substrate was added for 25 min, and the reaction was stopped with 2 M H₂SO₄. Absorbance was measured at 450 nm. For negative control, either N protein or CRPs were omitted from the assay.

### Binding of CRPs on virions mediated through N protein

An ELISA was performed to determine whether the N protein can recruit soluble CRPs to the SARS-CoV-2 virion. In short, 10 µL of medium from the infected cells, concentrated by ultrafiltration spin column, containing 1 × 10^9^ PFU was deposited in the center of each well (96-well ELISA plate), allowed to dry, and then 100 µL of 4% paraformaldehyde was added and incubated for 10 min. The plate was washed with PBST-20 and blocked with 5% BSA in PBS for 1 h at room temperature. Simultaneously, 28 µl of N protein (50 µg) was mixed with 10 µl of NHS and preincubated for 1 h at room temperature. After incubation, the volume was adjusted to 5 ml with PBST-20 and 100 µl of this suspension was added to the wells with fixed virus. Wells were incubated for 1 h at room temperature and then washed three times with PBST-20. Primary CRP-specific antibodies (anti-C1 inhibitor polyclonal, anti-C4BP, anti-factor H antibody and anti-vitronectin diluted 1:500 (v/v) in 1% BSA) for 1 h with constant shaking (350 RPM). The wells were washed three times with PBST-20 and incubated with HRP-conjugated protein A/G diluted 1:5,000 (v/v) in 0.5% BSA for 1 h with constant shaking (350 RPM) at room temperature. Final washing was performed three times with PBST-20 and one time with PBS. After the final washing, Ultra-TMB ELISA substrate was added for 25 min, and the reaction was stopped with 2 M H_2_SO_4_. Absorbance was measured at 450 nm. For negative control, either the N protein or NHS was omitted from the assay.

### Concentration-dependent recruitment of the CRPs by N protein on the virion

The ELISA was performed as described in the previous section, except that NHS was preincubated with various concentrations of the N protein. In short, 100 µl diluted NHS (1:500 in PBST-20) was spiked with varying concentrations of protein N (2000 ng, 1000 ng, 500 ng, 250 ng and 125 ng) and preincubated for 1 h at room temperature. After incubation, the mixture was transferred to the wells in which the virus was fixed. Wells were incubated for 1 h at room temperature and then washed three times with PBST-20. All other steps of ELISA, i.e., incubation with anti-CRPs antibodies, secondary antibody, and detection with Ultra-TMB ELISA substrate, were as described above. The assay was performed in triplicate. As a negative control, N protein was omitted from the assay.

### Assessment of Inhibition of the formation of MAC complex on virions in presence of protein N

50 µl of diluted NHS (40% [v/v] in PBS) was spiked with varying concentrations of protein N (0.5, 1, 2 and 4 µg/mL) and preincubated for 1 h at room temperature. Virions purified with sucrose gradient were then added to the preincubated NHS and incubated at 37 °C for 30 min. After incubation, samples were placed on 30% sucrose cushion and centrifuged at 100 000 x g, 4 °C for 22 h (rotor S50A, Himac, Japan). After centrifugation, the supernatant was removed, and pellet resuspended in 50 µL of PBS. Samples were heat-inactivated at 65 °C for 15 min and used for dot blot to detect C5b-9 deposition. First, 5 µL of each sample was spotted on nitrocellulose membrane and left to dry. The membrane was blocked with 5% BSA in TBS for 1 h at room temperature and washed once with TBST-20. The membrane was then incubated with anti-C5b-9 antibody diluted 1:1000 (v/v) in 0.5% BSA in TBST-20 for 1 h with gentle shaking at room temperature. After incubation, the membrane was washed with TBST-20 three times and then incubated for 1 h with IRDye 800CW donkey anti-rabbit IgG (H + L) antibody diluted 1:15,000 (v/v) in 0.5% BSA in TBST-20. The membrane was washed three times with TBST-20 and scanned on Odyssey CLx at 800 nm (LiCor). As a positive control for C5b-9 deposition, virions were incubated in NHS without N protein. As a negative control (no C5b-9 deposition), virions were incubated in NHS containing 25 mM EDTA.

## Conclusion

Altogether our results show that SARS-CoV-2 N protein interacts with soluble CRPs in human serum and might act as a complement evasion protein, recruiting CRPs on the surface of SARS-CoV-2 virions. We report on a possible complement evasion mechanism where viral protein (N) recruits soluble CRPs on the surface of virion via interaction with its main antigen (S). More research is needed, however, to determine the significance of SARS-CoV-2 interactions with CRPs in modulating the complement system.

## Supplementary Information

Below is the link to the electronic supplementary material.

Supplementary Material 1

## Data Availability

Data generated in this study are available from the corresponding author upon reasonable request.
