# Supplementary material for: SARS-CoV-2 nucleocapsid protein forms complexes with soluble complement regulatory proteins that can bind to the virion
Source: Sci Rep. 2026 Jan 29;16:6599. doi: 10.1038/s41598-026-37866-4 (PMC12913989; doi:10.1038/s41598-026-37866-4)
Supplement: Supplementary file 1 — Supplementary Material 1 [file 41598_2026_37866_MOESM1_ESM.pdf]

Supplementary information

SARS-CoV-2 nucleocapsid protein forms complexes with soluble complement regulatory proteins that can bind to the virion

Jakub Víglaský[1], Katarína Bhide[1], Lea Talpasova[1], Ľubica Fialová[2] , Mangesh Bhide[1,2]

Supplementary information 1

a. Cloning of the synthetic gene encoding spike protein, production and purification of recombinant protein

Synthetic genes were cloned into pCMV-based mammalian expression vector and the plasmid DNA was amplified from transformed *E. coli* DH5α strain (ThermoFisher Scientific, Slovakia) grown overnight at 37°C in LB (Luria-Bertani) medium (Sigma-Aldrich, USA) supplemented with kanamycin (25 µg/mL; Duchefa Biochemie, Netherlands). Plasmid extraction and purification were performed using PureLink HiPure Plasmid Filter Maxiprep Kit (ThermoFisher Scientific). Next, the purified plasmid (100 µg/mL) was electroporated into 2.1 x 10^8 cells/mL of human cell line Expi293 resuspended into electroporation buffer using MaxCyte STX (MaxCyte, MD, USA). After 30 min of resting time, the electroporated Expi293 cells were diluted to obtain the final concentration of 3.1 x 10^6 cells/mL and incubated with shaking at 37°C for 24h. Thereafter, the culture was incubated for about 4-6 days at 32 °C thereby reducing the cell viability to 50% of initial concentration. The reduced culture was centrifuged at 300 x *g* for 12 min and the resulting cell culture medium containing secreted recombinant protein was stored at -20 °C until further purification on ÄKTA purifier (GE-healthcare, Chicago, USA). In short, the cell culture medium was re-centrifuged at 20,000 *x g* and the clarified supernatant was added with 0.5 M NaCl (Sigma-Aldrich, USA) and filtered through 0.2 µm membrane filter. The filtrate was subjected to Nickel affinity chromatography on ÄKTA purifier using 5 mL Cytiva His-Trap™ affinity column (ThermoFisher Scientific). After stringent washing with 4 column volume of 20 mM sodium phosphate buffer (pH 7.4), the His-tagged protein was eluted by 0.5 M imidazole in 20 mM sodium phosphate buffer (pH 7.4). Thereafter the eluted protein was further purified on Strep-Tactin® affinity purification system (5 mL Strep-tag II column; IBA GmbH, Germany) using 10 mM desthiobiotin elution solution. Lastly, the purified recombinant spike was subjected to HiTrap™ Desalting column (GE Healthcare) to perform buffer exchange into Phosphate buffer saline (PBS, pH 7.4) and filter sterilized using 0.2 µm syringe filter. The recombinant spike protein contains residues from T^{19} to O^{1208} (GenBank:MN908947). Mutations K^{98}-P^{986}, V^{987}-P^{987} were introduced to achieve perfusion stability and furin cleavage site was substituted with $GSAS^{682–685}$). Recombinant Spike also contained T4 fibritin trimerization motif, HRV3C protease cleavage site, 8x his-tag and a twin-Strep-tag at the C-terminus (Figure S1)

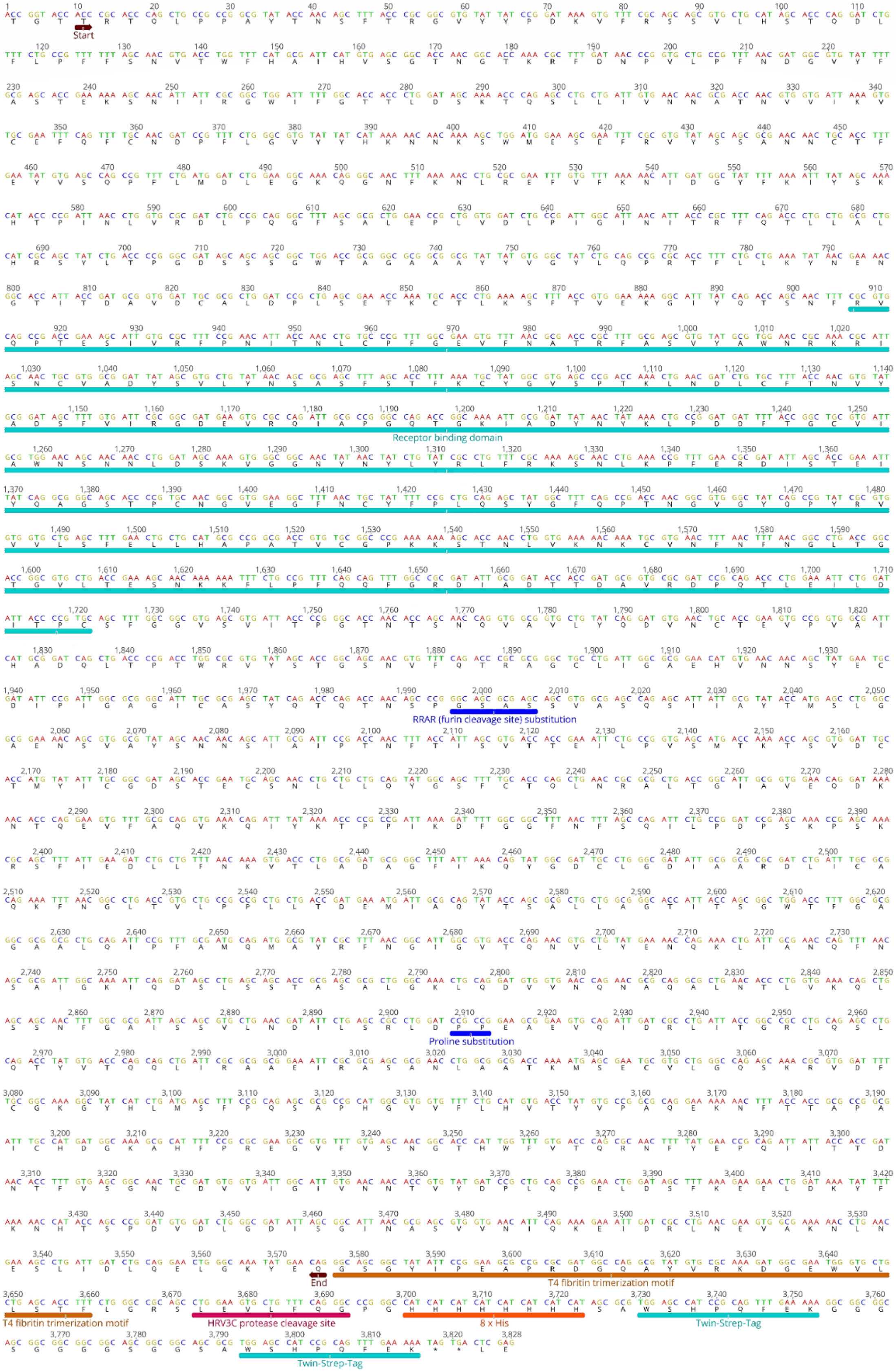

Start
Receptor binding domain
RRAR (furin cleavage site) substitution
Proline substitution
End
T4 fibritin trimerization motif
T4 fibritin trimerization motif
HRV3C protease cleavage site
8 x His
Twin-Strep-Tag
Twin-Strep-Tag

Figure S1 - Nucleotide and its corresponding amino acid sequence of spike protein (GenBank: MN908947) with marked annotations for – start and end of target sequence, receptor binding domain (underlined), furin cleavage site substitution, proline substitution (for perfusion stability), T4 trimerization motif, HRV3C protease cleavage site, 8 x His (histidine tag), and twin step tag (streptavidin tag).

b. Quality control of the recombinant protein by LDS-PAGE and MALDI

The protein sample was mixed with lithium dodecyl sulfate sample buffer (4X LDS sample buffer, Invitrogen, Slovakia) as per manufacturer's instructions and incubated at 72°C for 10 min. Electrophoresis was carried out at 200V in 1X running buffer (20X NuPAGE MOPS SDS running buffer, Invitrogen , USA until the dye reached the bottom of the gel (12% Bis Tris polyacrylamide 12 well gel, Invitrogen). Proteins were stained with Coomassie staining (Bio Rad). For MALDI TOF MS, 0.8 µl of the purified protein was mix ed with 0.8 µl sDHB matrix (2,5 2,5 dihydroxybenzoic acid and 2 hydroxy 5 methoxybenzoic acid, Bruker Daltonics, Germany) dissolved up to saturation in TA50 (50:50 [v/v] acetonitrile:0.1% trifluoroacetic acid, TFA, Sigma). One microliter of the protein matrix mix was spotted on the ground steel plate (Bruker Daltonics). The acquisition was performed in flexControl (V 3.4, Burker Daltonics) in linear mode with 60 Hz laser intensity (200 shots) on Microflex with reflectron MALDI mass spectrometer (Bruker Daltonics). Mass was analyzed in flexAnalysis V3.4 software of Bruker Daltonics by comparing it with calibrants (Protein calibration kit I, Bruker Daltonics).

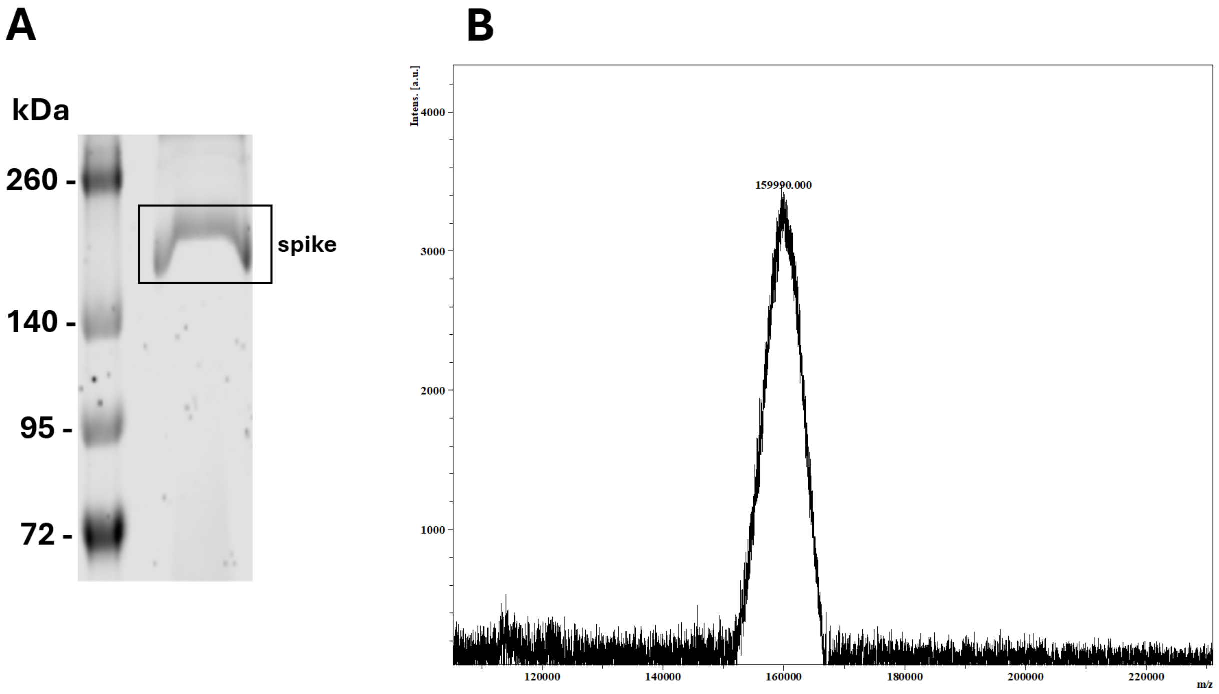

Figure S2 - A - LDS-PAGE of recombinant spike protein **B** - MALDI-TOF MS spectrum of recombinant spike protein. The predicted molecular mass of spike protein was ~160 kDa.

Supplementary information 2

Table 1. Antibodies and proteins used in experiments

Antibody	Manufacturer	Catalog number
SARS-CoV-2 Nucleocapsid Protein Human Monoclonal Antibody [clone: HC2003]	GenScript Biotech	GENSA02039
SARS-CoV-2 Spike Protein (RBD) Antibody	Thermo Fischer Scientific	T01KHu
Anti-C1 inhibitor polyclonal	Santa Cruz Biotechnology	sc-46298
Anti-C4BP (Used in ELISA)	Abcam	ab200345
Anti-C4BP polyclonal (used for purification of C4BP)	Santa Cruz Biotechnology	sc-17216
Anti-Factor H antibody [OX-24]	Abcam	ab118820
Anti-vitronectin [EP873Y] (used in ELISA as well as for purification of VNT)	Abcam	ab45139
Anti-C5b-9 antibody	Abcam	ab55811
IRDye 800CW Donkey anti-Mouse IgG (H + L)	Li-cor	926-32212
IRDye 800CW Donkey anti-Rabbit IgG (H + L)	Li-cor	926-32213
IRDye 800CW Donkey anti-Goat IgG (H + L)	Li-cor	926-32214
His probe HRP	PIERCE	15165
Anti-mouse IgG Goat Polyclonal Antibody (HRP)	ImmunoReagents	GTXMU-003-L2HRPX
Anti-human IgG (HRP)	Abcam	AB6759
Protein A/G - HRP conjugated	Thermo Fischer Scientific	32490
**Protein**	**Manufacturer**	**Catalog number**
SARS-CoV-2 spike protein	See supplementary information 1	Produced in this study
SARS-CoV-2 - nucleocapsid protein 48.3 kDa	Jena Bioscience	PR-1454
Complement Factor H from human plasma	Sigma-Aldrich	C5813
C1 Esterase Inhibitor	Merck	E0518
C4BP	-	purified in this study
Vitronectin	-	purified in this study

Supplementary information 3

Virus titration (plaque assay)

Vero E6 cells (Sigma-aldrich, USA) were seeded in 24-well plate (100,000 cells/well, 36th passage) and cultivated at 37°C, 5% CO_2 in DMEM (Sigma) supplemented with 5% fetal bovine serum (Biowest, France), 2mM L-glutamine (Serana, Germany), 50 U of penicillin and 50 µg/mL of streptomycin (Jena Bioscience, Germany). At 80% confluency, the medium was discarded and 200 µL of fresh complete DMEM was added to wells.

Meanwhile, virus containing cell culture medium was diluted with fresh complete DMEM as depicted below (Figure S3). The cells were then infected with 100 µL from each dilution and incubated for 1 hr at 37 °C, 5% CO_2. For virus control, the cells were infected with 100 µL of concentrated virus-containing medium with known titer (4.92 x 10^5 $TCID_{50}$/mL). 100 µL of complete DMEM without virus was used as cell control. Every dilution, as well as controls were in duplicates. After incubation, 500 µL of 1.5% carboxymethyl cellulose (mixed with fresh complete 2x DMEM (1:1, v/v) were added to each well. The plate was incubated for 72 hrs at 37°C, 5% CO_2. After incubation, 400 µL of 8% formaldehyde was added to each well and incubated at room temperature room for 30 min. Wells were washed with water and the plate was submerged in 100% methanol for 5 min for disinfection. Staining was performed using 500 µL of crystal violet for 15 min. Wells were rinsed with tap water and dried at room temperature. Plaques were manually counted (Figure S4), and PFU/mL were calculated. The titter was 1 x 10^{12} PFU/mL.

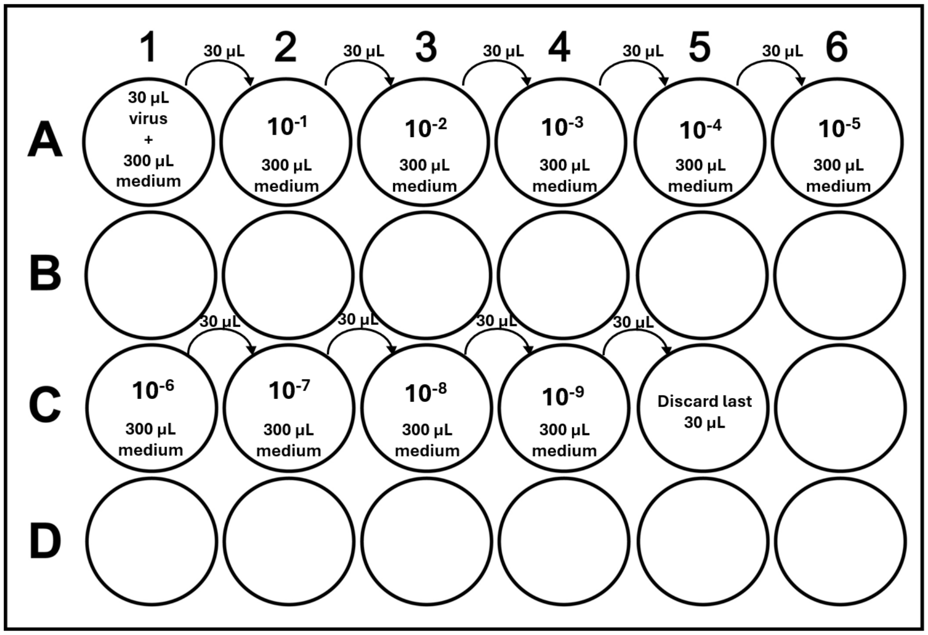

Figure S3 – Schematic representation of the 10-fold dilution of cell culture medium containing virus used for virus titration

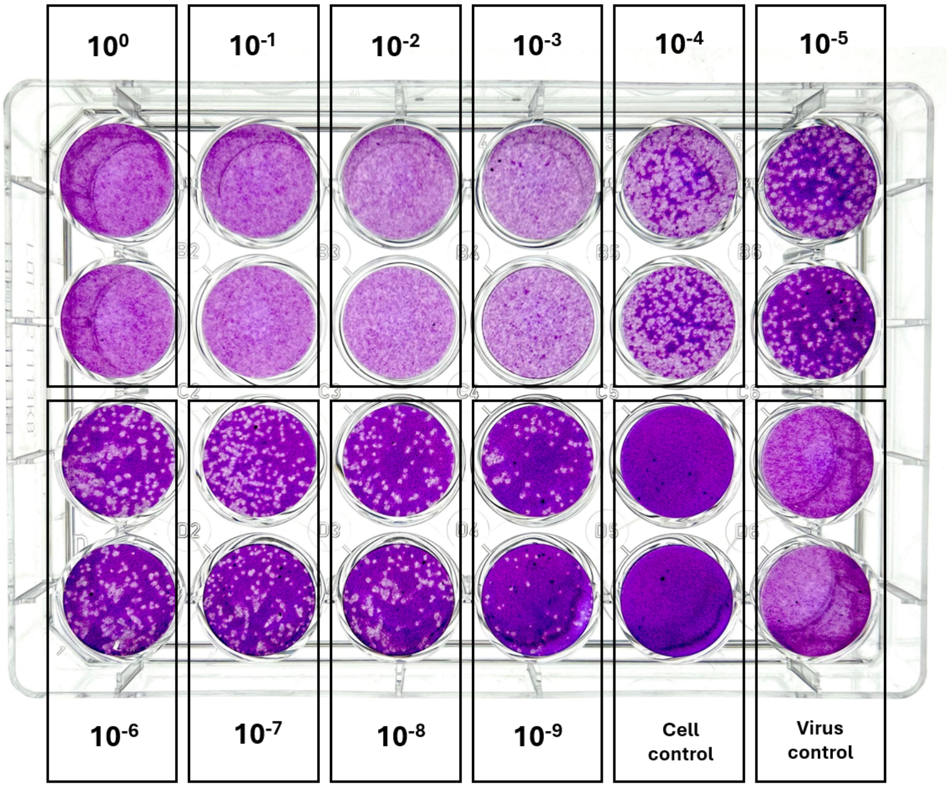

Figure S4 – Virus titration in 24-well plate after staining.

Supplementary information 5

Original scans of membranes used to generate figures

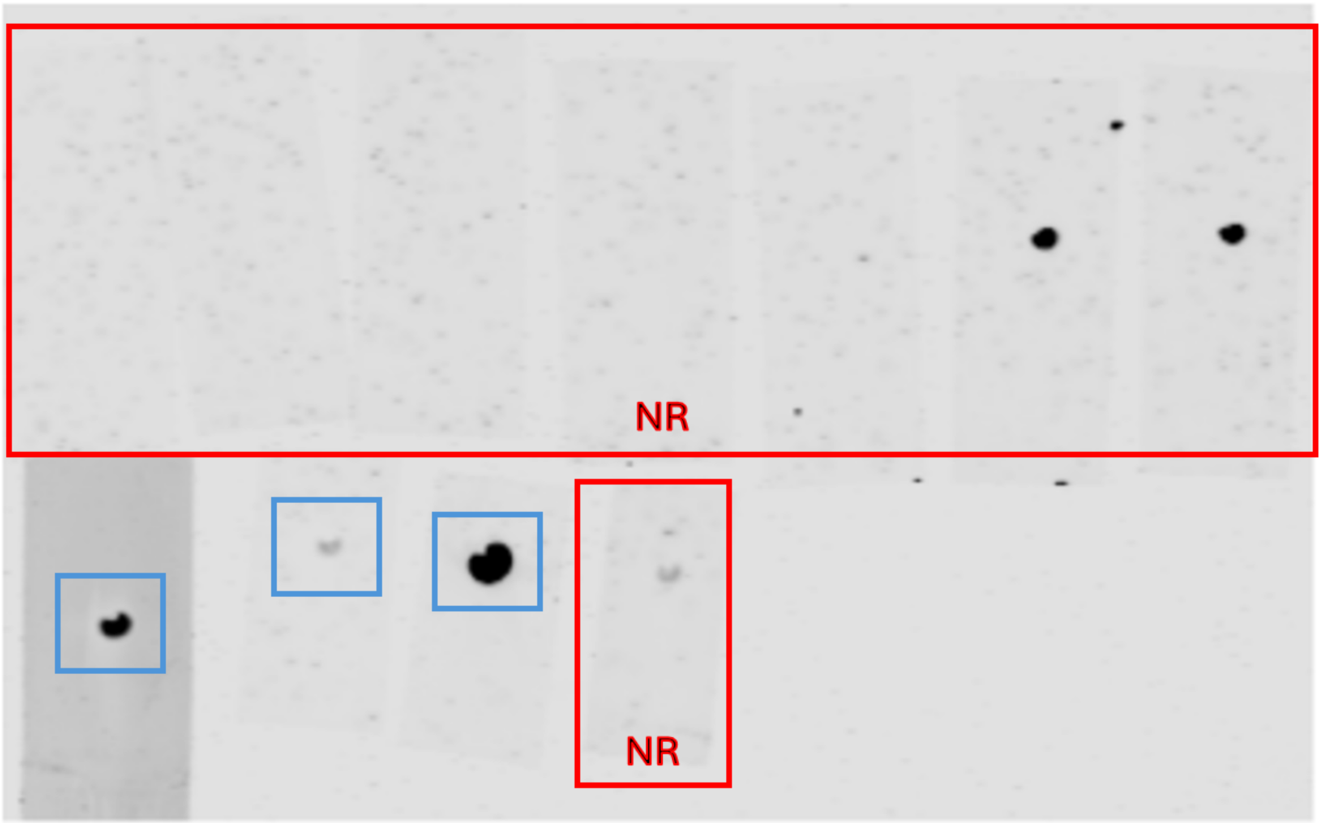

Figure S6 – dot blot nitrocellulose membranes used to generate Figure 1, Panel A.

NR – not related to this manuscript

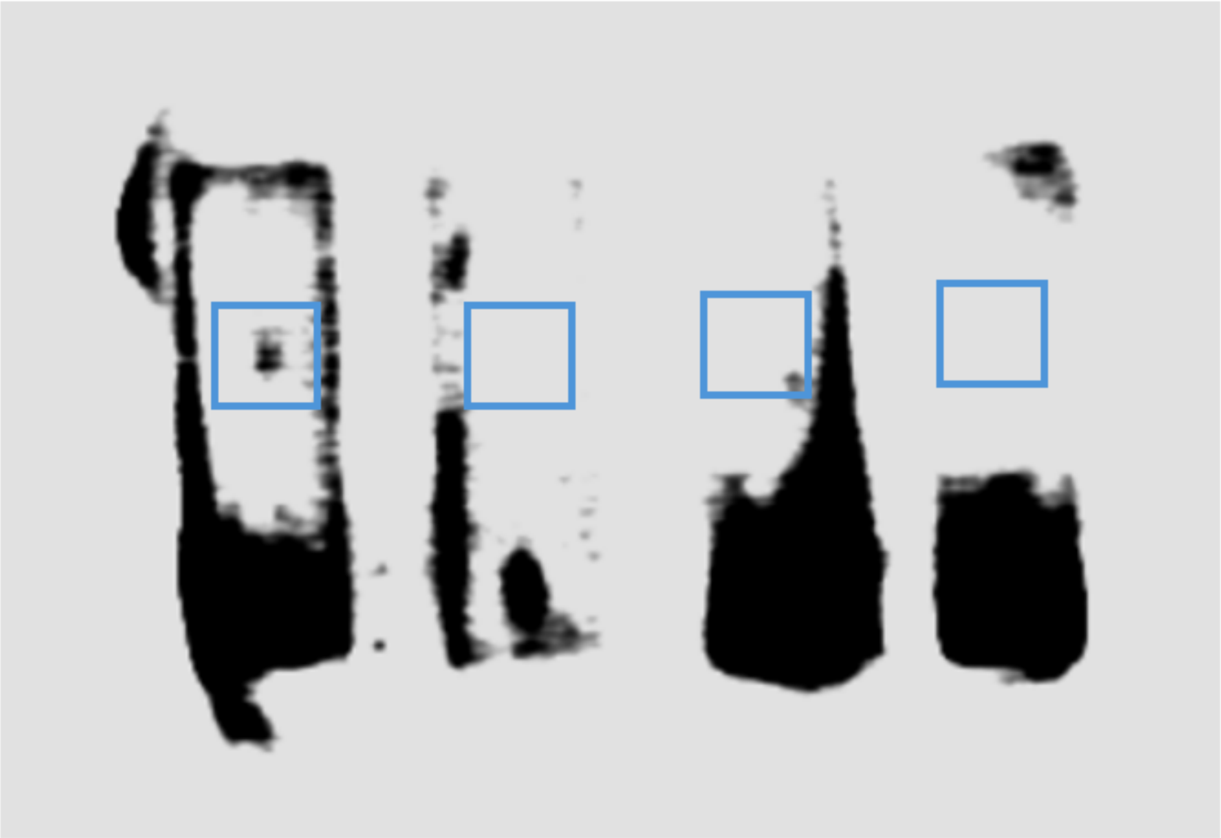

Figure S7 – dot blot nitrocellulose membranes used to generate Figure 1, Panel B.

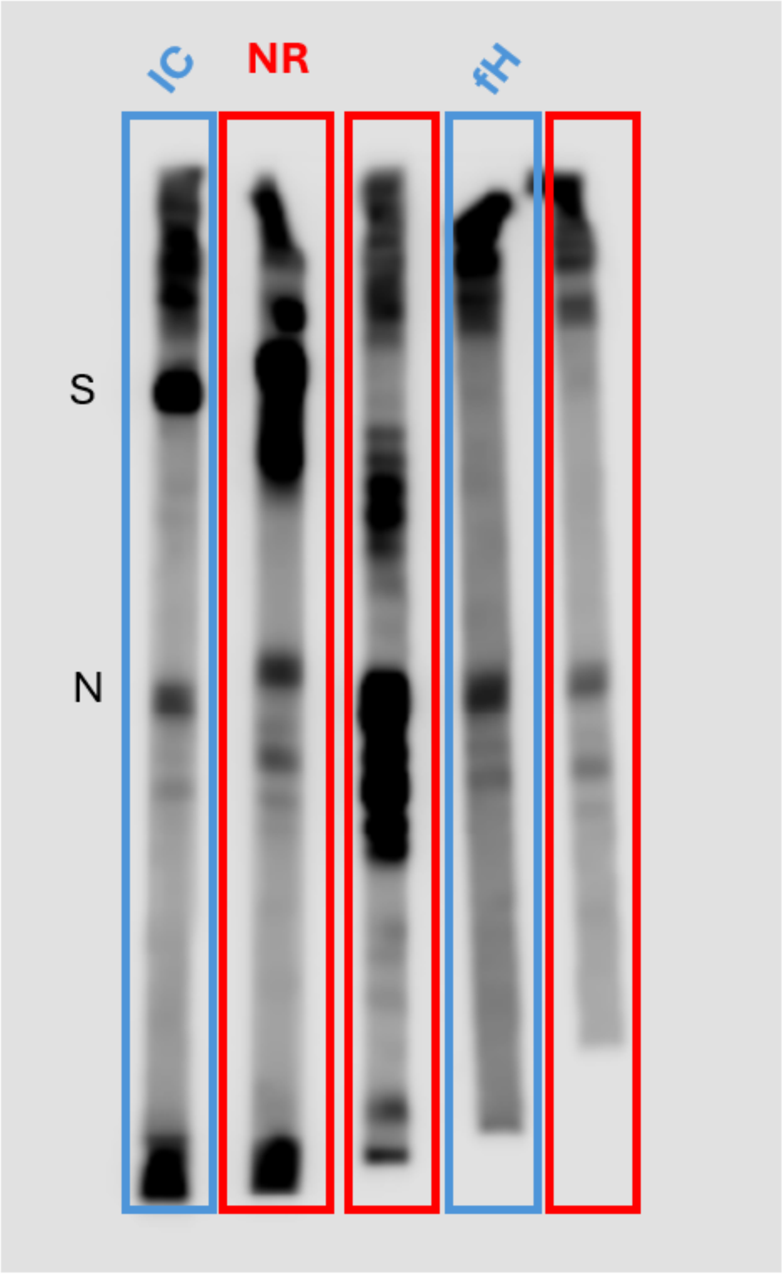

Figure S8 – Far Western blot PVDF membranes used to generate Figure 4, Panel A.

S – SARS-CoV-2 spike protein

N – SARS-CoV-2 nucleocapsid protein

IC – input control

NR – not related to this manuscript

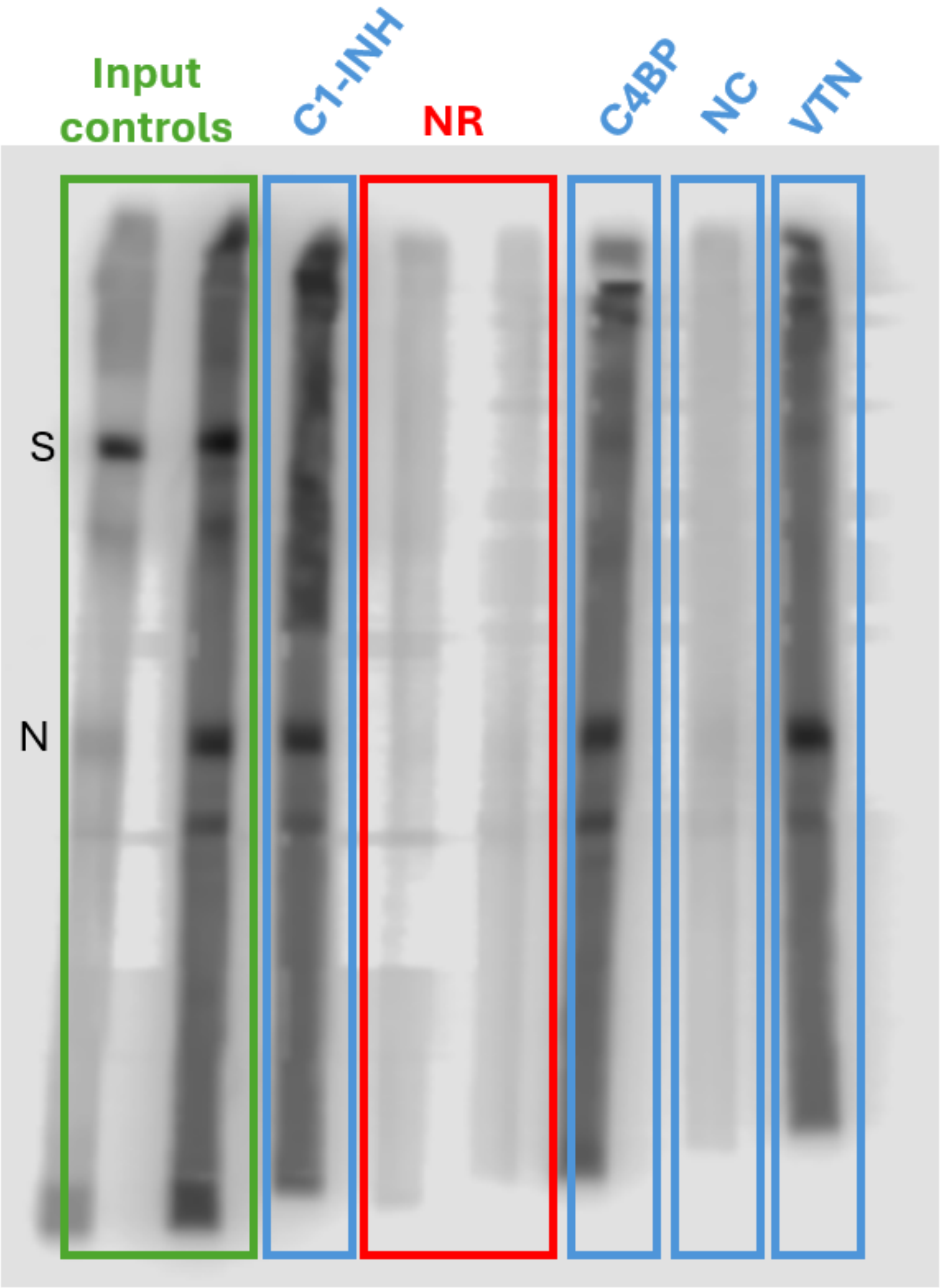

Figure S9 – Far Western blot PVDF membranes used to generate Figure 4, Panel A.

Input controls were used for identification of interactions, not to generate Figure 4, Panel D.

NC – negative control

NR – not related to this manuscript

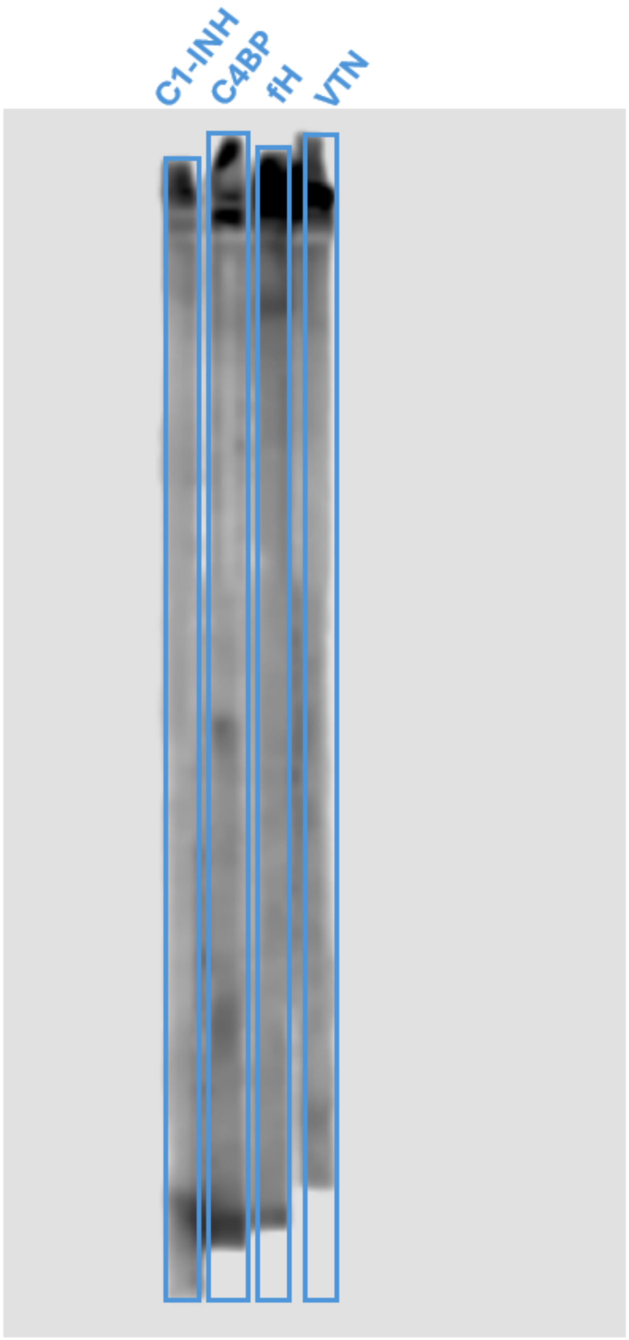

Figure S10 – Far Western blot PVDF membranes used to generate Figure 4, Panel A.

These membranes were prepared in the same manner as membranes in Figures S7 and S8, except that no renaturation was performed prior to incubations.

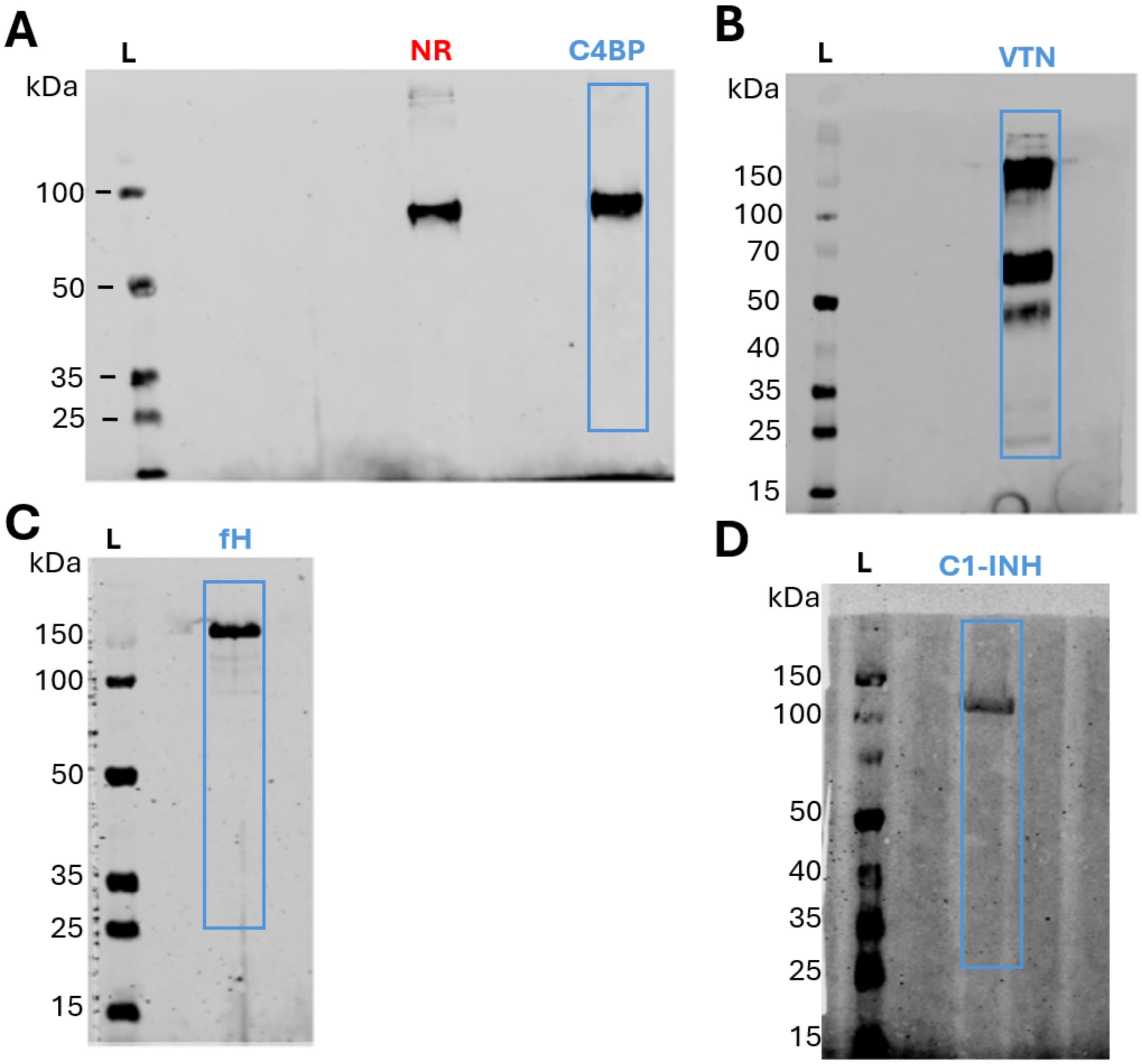

Figure S11– Original scans of nitrocellulose membranes used to generate Figure 4, Panel D.

A – Western blot used to detect C4BP

B – Western blot used to detect vitronectin (VTN)

C – Western blot used to detect factor H (fH)

D – Western blot used to detect C1 inhibitor (C1-INH)

L – ladder (marker)

NR– not related to experiments of this paper

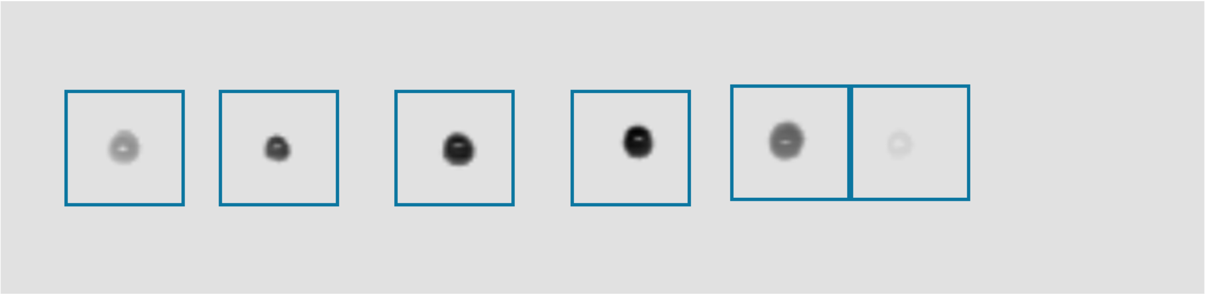

Figure S12– Original scan of nitrocellulose membrane used to generate Figure 5, Panel D.
